# Medication adherence in pet bird owners: index development and assessment of various application types via an online questionnaire

**DOI:** 10.1186/s12917-026-05457-0

**Published:** 2026-04-14

**Authors:** Felica Fontaine, Katrin Drasch, Anne-Kathrin Burmeister, Sebastian Prechsl, Christoph Müller, Rüdiger Korbel, Monika Rinder, Nicole J. Saam

**Affiliations:** 1 Clinic for Birds, Small Mammals, Reptiles and Ornamental Fish, Center for Clinical Veterinary Medicine, Ludwig-Maximilians-Universität München, Oberschleißheim, Germany; 2 Institute of Sociology, Friedrich-Alexander University Erlangen-Nürnberg, Erlangen, Germany; 3 present address: Office of Academic Affairs, Veterinary Faculty, Ludwig- Maximilians-Universität München, München, Germany; 4 present address: Institute for Employment Research DE, Nürnberg, Germany

**Keywords:** Medication adherence, Bird owner, Avian medicine, Adherence index, Compliance, Veterinary practice

## Abstract

**Background:**

Adherence to medication prescriptions substantially contributes to therapeutic success. In pet bird medicine, however, measures to determine adherence and possible causes are lacking. This study aimed to develop medication adherence indices for companion bird owners, including single and multiple application types.

**Results:**

The single veterinary medication adherence index (SVMAI-4), a 4-point index developed for individual treatment types, considers changes in treatment type, dosage or treatment time, early discontinuation, and total refusal for 9 application types. The overall adherence index OVMAI-5, a 5-point index addresses multiple application types and also considers nonprescribed medication. An online questionnaire was developed to evaluate the indices and measure determinants of adherence based on self-reports and implemented in a cross-sectional survey of 1480 bird owners. The results revealed that oral application directly into the beak (62.5% of birds) and by drinking water (28.3%) were the most common application types. Distinct differences among application types were found. With a range of 0 (full adherence) to 4 (full nonadherence), adherence was highest for cutaneous application (mean = 0.36, standard deviation (SD) = 0.736) and lowest for peroral application into the beak (mean 0.59; SD = 1.023) and injection (mean 0.73, SD = 1.126). The OVMAI-5 calculated when assessing the use of multiple application types resulted in a mean score of 0.84 (SD = 1.247), ranging from 0 to 5.

**Conclusions:**

The results suggest a need for action regarding the application types most commonly used in avian medicine. Wherever possible, oral administration via the beak should be avoided. Due to the self-reporting method used, it must be assumed that our results overestimate adherence and cannot be externally evaluated in a straightforward way. Animal patient-related factors are the most important determinants of medication adherence.

**Supplementary Information:**

The online version contains supplementary material available at 10.1186/s12917-026-05457-0.

## Background

 Adherence to medication was defined as the extent to which a person’s behavior—taking medication, following a diet, and/or executing lifestyle changes—corresponds with agreed-upon recommendations from a health care provider [1], and it has been described as a process by which patients take their medications as prescribed [2]. Nonadherence is a common but often underestimated problem in human and veterinary therapy. In veterinary practice, patient owners do not adhere to the prescribed medication plan and risk only limited or no therapeutic success.

The term adherence was preferred over the term compliance, defined as the extent to which a person’s behavior (in terms of taking medication, following diets, or executing other lifestyle changes) coincides with medical or health advice and does not include the important aspect of the patient or patient owner’s agreement with medication, thus following common recommendations [2].

There are various definitions of medication adherence for human patients or animal keepers. In some studies, any deviation from the prescribed medication was regarded as nonadherent behavior [3–7]. Others used a defined rate of prescribed doses, mostly 80%, as a cutoff to identify a patient owner as adherent or nonadherent, on the basis of the assumption that this cutoff might reflect clinical relevance [8–12]. Both procedures result in dichotomizing adherence, and data are reported as the overall mean or median adherence of the group of patients or keepers [8, 10]. According to a commonly used definition of adherence as the extent to which prescribed medication is followed, individual adherence can also be described quantitatively as the percentage of prescribed doses apparently given according to pill counts or as reported by patients, the percentage of days on which the correct number of doses were given, or the percentage of doses that were given on time [8, 10]. Some adherence measures combine these different aspects to calculate adherence values or categorize persons into different groups, for example, high, medium or low adherence [13].

It is well known in the field of human medicine that suboptimal and thus inadequate adherence to drug therapy can lead to increased morbidity, mortality and costs for the health care system [1, 12, 14], and nonadherence is regarded as a major problem worldwide [1]. According to adherence report reviews, an average rate of adherence by patients to treatment recommendations of 50–60% has been reported, which falls to 30% for complex treatments [1, 15, 16]. Adherence rates range from 5 to 96%, depending on the measuring instrument, the definitions used for adherence and nonadherence, the disease examined, the prescribed therapy and the environment in which adherence was recorded [17].

In the field of veterinary medicine, medication adherence and nonadherence must be assumed to be of similarly high relevance. Overall, medication adherence rates of 64% and ranges of 20–73% have been reported, which are reduced to 20–30% for certain treatments [18–21]. In contrast to the situation in human medicine, however, there is little research in the field of veterinary medicine [3, 18, 21, 22] and there is no scientific literature on medication adherence in avian medicine, particularly regarding pet birds. So far, the focus has been on dogs and cats, and on oral medication using pills. In a systematic review published in 2018 and assessing the existing published evidence base concerning factors that influence the medication adherence of cat and dog owners, only eight publications were found, mostly related to short-term oral antibiotic therapy [23]. To the best of our knowledge, there has been no such investigation in the field of companion birds thus far, where medication types are extremely variable. Here, medication is generally not based on giving pills (probably because of the small and varying size of the patients and lacking availability of appropriate pill sizes) but, most frequently, is based on giving drugs in liquid form orally via the beak or on medication by drinking water or feed.

In human medical research studies and meta-analyses, a wide range of direct and indirect adherence measurement methods have been developed on the basis of objective methods such as pill counting, electronic monitoring and electronic healthcare databases as well as subjective measures such as self-reports [10, 24–27]. Gold standards for both objective and subjective measures, however, do not yet exist. As reviewed by Nguyen, La Caze [10], subjective measures differ from objective measures in that they have the potential to identify the specific reasons for a patient’s nonadherence, can be simple to use and have low costs. The main disadvantages of these methods are that they are prone to recall errors and social desirability bias [10].

There is, thus far, no measure to determine the medication adherence of animal owners in general use. The triadic relationships between veterinarians, animal patients and animal owners differ from the dyadic relationships between medical doctors and human patients. Some similarities were found only in pediatric medicine or in human relations, where a caregiver takes over responsibilities for the medication of a family member [8, 28]. Medication adherence measures used in human medicine such as the Medication Adherence Report Scales (MARS-5 and MARS-10) [29], the Malaysia Medication Adherence Assessment Tool (MyMAAT) [30], the Brief Medication Questionnaire (BMQ) [31], the Intentional Nonadherence Scale (INAS) [32] and the four-item Morisky Medication Adherence Scale (MMAS-4) [13] (but see [33]) thus cannot easily be transferred to companion animals, especially to birds [34].

To be highly valid, a measure should contain exactly defined factors. It has been recommended for humans that the measure should refer to one or more specific medications and recommended dosages. The therapy phase investigated (initiation, implementation, or discontinuation according to Vrijens, De Geest [2]) should be addressed and measured separately [25, 33]. Furthermore, depending on whether the study aims at determining the behavior of the patients during therapy or at detecting the causes of adherence or nonadherence, items should be appropriately selected. The latter aim might differ between unintentional and intentional nonadherence or determine possible outcome measures [26, 29, 33].

According to the World Health Organization [1], adherence is a multidimensional phenomenon determined by the interplay of five sets of dimensions, of which patient-related factors are just one determinant. Further dimensions, e.g., socioeconomic factors, factors related to the condition of the disease, factors related to the healthcare team/health system and factors related to treatment, should also be taken into consideration [1].

This study aimed to measure the degree of adherence to medication as well as possible reasons for the nonadherence of bird owners who were supposed to treat their bird at home. Therefore, veterinary medication adherence indices were developed to allow the adherence of bird owners to prescribed single medication application forms to be measured and, in cases where multiple medical application types had been prescribed, to allow overall medication adherence to be measured. The newly developed indices were then tested via an online questionnaire based on self-reports of pet bird owners.

## Methods

### Key features of the study design

A multistep process was used to develop the veterinary medication adherence indices OVMI-5 (overall veterinary medication adherence index) and SVMAI-4 (single veterinary medication adherence indices). Several scales and indices developed for humans (see above) have been reviewed and adapted. Next, questions and items for a questionnaire were developed to cover relevant information on pet bird owners and their bird patients. The final questionnaire, including the indices, was tested in an online survey (cross-sectional study) on the basis of self-reports of bird owners, and the data were analyzed.

### Construction of adherence indices and questionnaire design

To measure veterinary medication adherence on an index, a questionnaire was designed using a four-step procedure. The questions and items for the indices and the questionnaire were developed in a focus group by an interdisciplinary team of four veterinarians and one sociologist, all of whom were familiar with human‒bird relationships in professional or private capacity, thus following an approach described previously [35]. The data were collected in Germany; therefore, all the items and questions were prepared in German. An English translation of the questionnaire is available in the additional file 1.

#### First step

The basic requirements for an adherence index were as follows: For veterinary practice, we needed an overall index for a pet bird undergoing treatment, taking into account all the treatment types used for this individual. For research and causal analysis, we needed single indices for each type of pet bird treatment. Following Franke, Jagla [36] from human medicine and Kostka and Bürkle [37] from veterinary medicine, we distinguished seven dimensions of nonadherence (change in the type of treatment, total refusal, early discontinuation, change in dosage, change in the time of treatment, administration of additional nonprescribed medication, and selectivity in cases where multiple medical application types had been prescribed) and nine types of treatment (drinking water application (medication applied to drinking water), feed application (mixed into the feed), oral application (application straight into the beak), injection, cutaneous application, inhalation, bandage change and control, change of feed and/or change of husbandry). For some treatments, a single dimension of nonadherence may not apply, e.g., if no medication has been given with “bandage change” therapy, the change in dosage was irrelevant and had to be removed from the index construction. We gave all types of treatment equal weights because we did not know whether the veterinarian had previously prioritized some treatment in the prescription. Similarly, we did not prioritize some dimensions of nonadherence because it was not possible to judge how important a dimension was relative to other dimensions.

#### Second step

Drafts of an overall index and single indices were formalized. The construction of the index was implemented with a method that is generally referred to as “index formation” or the “point total method” [38]. For each index to be constructed, the maximum number of points (or 100%) should denote the state of full nonadherence to therapy, 0 points (or 0%) should denote the state of full adherence to therapy, and intermediate numbers of points should reflect partial adherence. The single adherence index was an additive index calculated from four dimensions of nonadherence: change in the type of treatment, early discontinuation, change in dosage, and change in the time of treatment, unless the bird owner reported total refusal, amounting to a 4-point index. The overall adherence index was an additive index calculated from the mean value of all single adherence indices pertaining to one case of a pet bird patient and supplemented on the one hand by a score for the administration of additional nonprescribed medication and, on the other hand, for selectivity in cases where multiple medical application types had been prescribed (because both can be observed only once for each bird patient), amounting to a 6-point index.

#### Third step

Data were collected in a cross-sectional study via an online survey, and empirical adherence was measured via the drafted indices. A questionnaire developed for this purpose was used to evaluate the instruments. The questionnaire was divided into eleven sections, which covered the following topics: the participant’s sociodemographics (such as gender, age, education); the history of bird ownership; information about the patient’s bird (species, age, sex); details on the reasons for veterinary treatment, the type(s) of treatment and adherence/nonadherence to the prescribed therapy; determinants of adherence derived from the World Health Organization [1] (29 items measuring six determinants: patient owner-related factors, veterinary care-related factors, disease-related factors, therapy-related factors, patient-related factors, socioeconomic factors); and the human‒bird relationship was measured via the “owner‒bird relationship scale” (OBRS) [35]. An English version of the questionnaire is included in the additional file 1.

Before the survey was finally launched, it was subjected to a pretest. The participants provided information about the content of the questionnaire concept and the technical implementation of the survey. The pretest ran for a total of six days, and fifteen participants took part. The preliminary testers included two members of the panel of experts who reviewed the scientific aspects and had a general overview of the project and its development. Furthermore, eight bird owners, who evaluated the content and time needed to complete the survey, were among the testers. The third group comprised people who were asked to check their language, time, technical conditions, and layout. Each of the test subjects used a comment function implemented in the online questionnaire or gave feedback via telephone. Spelling errors, technical errors, and layout problems could thus be identified and corrected. With this procedure, we additionally ensured that the questions were understood equally by all the participants. The average answer time of the pretesters was approximately 30 min.

#### Fourth step

**Fig. 1:**
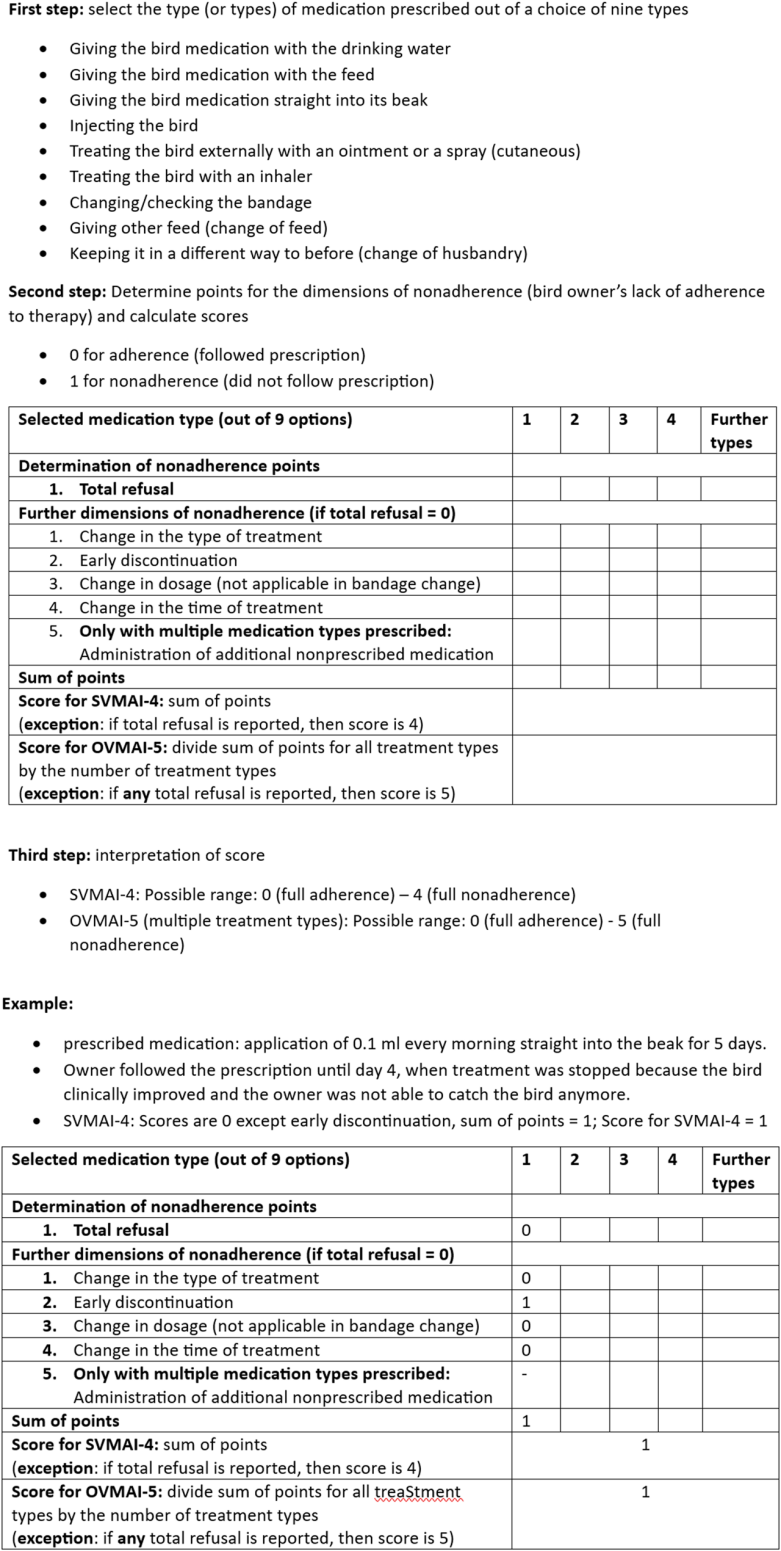
Adherence score determination using the SVMAI-4 or OVMAI-5

In the final step, the draft indices were revised, and empirical adherence was measured according to the revised indices. The use of indices generated from several items is a standard procedure in empirical research [39, 40]. The draft indices calculated from the empirical data revealed two major problems that resulted in cases in which multiple medical application types had been prescribed: (i) how total refusal of one treatment, and (ii) how selectivity (bird owners agreed with “yes” on this item: “If you had to give more than one medicine: I have omitted at least one medicine”) was evaluated. As our data were based on self-reports by bird owners, we had only limited knowledge of the prescribed therapy. In particular, we do not know how relevant one treatment or one medicine is for the overall success of the therapy. For (i) total refusal, we revised our decision from step one to give all types of treatment equal weights and not to prioritize some dimension of nonadherence. Instead, we decided to systematically assume the worst case, i.e., the refused treatment was assumed to be the most important of all treatments for that bird patient case. This meant that we prioritized total refusal in both the overall and the single adherence indices: if the patient’s owners had completely refused one treatment, the maximum score was assigned to this treatment’s single index as well as the overall index. In both cases, this represented the complete lack of medication adherence. For (ii) selectivity, we recognized that only a minority of bird owners had been guided by the filter system to this item (*N* = 624), i.e., those bird owners who had actually indicated multiple types of medication before. Consequently, only for these 624 cases would it have been possible to calculate the overall adherence index. To calculate the overall index for most pet bird cases in our dataset, we decided to remove this selectivity from the dimensions included in the overall index. Thus, the revised overall veterinary medication adherence index was a 5-point index (OVMAI-5), and all single veterinary medication adherence indices were 4-point indices (SVMAI-4). Individual adherence scores were calculated as illustrated in Fig. 1 and according to the following equations.

Let *x*_*ijk*_ denote a prescribed treatment *i* (*i* = 1 … 9) of a pet bird patient *j* (*j* = 1 … *n*) by pet bird owner *k* (k = 1 … m), and let *v* denote the overall number of treatments *i* prescribed for pet bird patient *j*.

Let w_l_ (*l* = 1 … 4) denote four possible nonadherent ways of behavior and *r* total refusal (*r* ϵ [0, 1]). In the therapy applied by the pet keepers, they received 0 points for showing adherent behavior, 1 point for nonadherent behavior and 4 points for total refusal.

The adherence score SVMAI-4 (single veterinary medication adherence index) for the treatment *i* of pet bird patient *j* by pet bird owner *k* (with 0 < = SVMAI-4_ijk_ < = 4) is defined as:

1 $$\mathrm{SVMAI}-4 _{ijk} = 4\; \mathrm{if} \left( \mathrm{r} _{ijk} = 1 \right) \mathrm{else} \sum \nolimits ^4_{l=1} W _{ijk}$$

Let *a* denote an additional nonadherent behavior (a ϵ [0, 1]), i.e., the administration of additional nonprescribed medication that pet keeper *k* may show during the overall therapy of this bird patient *j*, w denotes four nonadherent ways of behavior and *r* the total refusal. Let *q* (with 0 < = *q* <= *9*) denote the number of treatments *i* that the bird keeper completely refused during the overall therapy. In the overall index, the pet keepers receive an average score for the treatments, which is increased by 0 points for showing adherent behavior in *a* or 1 point for nonadherent behavior in *a*. However, if the keeper has completely refused at least one treatment (q_jk_ > = 1), s/he receives 5 points. Thus, the adherence score OVMAI-5 (overall veterinary medication adherence index) for the overall therapy of a pet bird patient *j* by pet bird owner *k* (with 0 < = OVMAI-5_jk_ < = 5) is defined as:

2 $$\mathrm{OVMAI} - 5 _{\mathrm{jk}} = 5 \; \mathrm{if} \left(\mathrm{q} _\mathrm{jk} >= 1 \right) \mathrm{else} \left( 1/\mathrm{v} \left( \sum \nolimits^v_{i=1} SVMAI-4_{ijk} \right) +a_\mathrm{jk} \right)$$

with *v* denoting the overall number of treatments. The overall scores for all pet bird patients were obtained by calculating the mean of the individual scores for the overall therapy of all pet bird patients. An overview of all variables used in Eqs. 1 and 2 is given in Table 1.

**Table 1:** Variables used to calculate the adherence indices SVMAI-4 and OVMAI-5

Variable name	Definition	Range of values
SVMAI-4		
* i*	prescribed treatment	*i* = 1 … 9
* j*	pet bird patient	*j* = 1 … *n*
* k*	pet bird owner	*k* = 1 … m
* x*_*ijk*_	prescribed treatment *i* of a pet bird patient *j* by pet bird owner *k*	
* v*	overall number of treatments *i* prescribed for pet bird patient j of pet bird owner *k*	0 < = *v* <= *9*
* w*_*l*_	four possible nonadherent ways of behavior	*l* = 1 … 4
* W*_*ijk*_	sum of possibly nonadherent ways of behavior *l* of pet bird owner *k* with respect to a *single* prescribed treatment *i* of pet bird patient *j*	0 < = *w* <= 4
* r*_*ijk*_	total refusal of pet bird owner *k* with respect to a prescribed treatment *i* of pet bird patient *j*	*r* ϵ [0, 1]
OVMAI-5		
SVMAI-4 plus:		
* a*_jk_	additional nonadherent behavior: the administration of additional nonprescribed medication that pet keeper *k* may show during the overall therapy of this bird patient *j*	*a* ϵ [0, 1]
* q*_jk_	the number of treatments *i* that bird keeper *k* completely refused during the overall therapy of pet bird patient j	0 < = *q* <= *9*

### Data collection

The study was conducted as an online survey in Germany via the EFS Survey (Unipark & Questback GmbH, Cologne/Germany). Data were collected from December 2018 to March 2019. The survey link to the questionnaire of the main study was distributed to bird owners throughout Germany, who were contacted through several sources via snowball sampling. This nonprobability sampling technique was used to reach as many bird owners as possible because of the unavailability of any appropriate database, such as a list of registered bird owners in Germany. Participants were reached via the internet (social networks, internet forums, websites, and email discussion groups—all of them about birds) and conventional methods (veterinary clinics, zoo shops, bird journals, and in-person groups to acquaint bird owners with the project), with a request for cross-posting and forwarding the internet link starting the survey.

The target group included bird owners who were supposed to treat their bird independently at home in accordance with the recommendations of their veterinarians. Because the survey link was freely available, anyone who was aware of the link and had internet access could participate. Only bird keepers who indicated that they had treated a bird at home in the past six months completed the entire questionnaire, including the adherence indices.

**Fig. 2:**
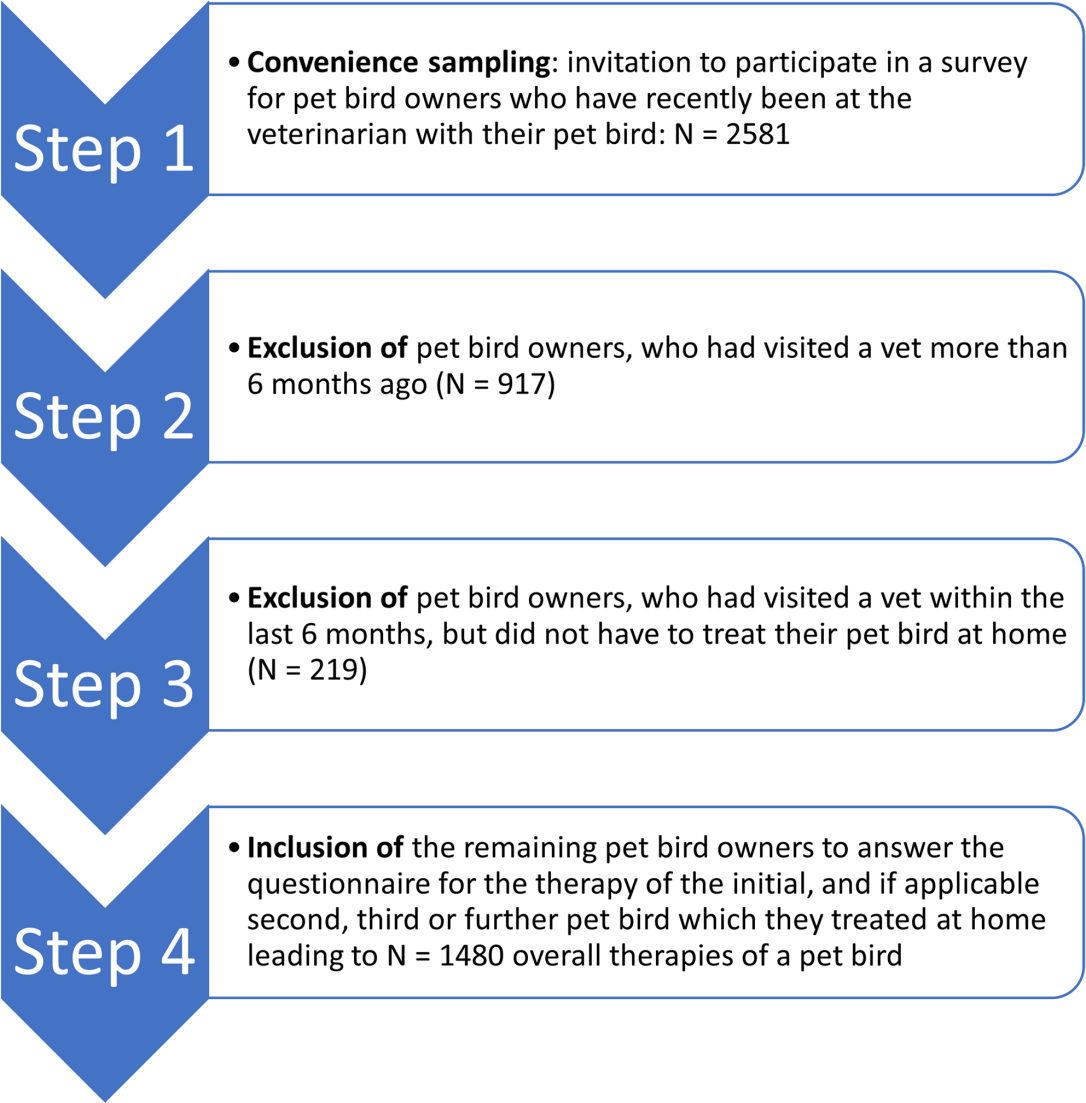
Overview of the data collection process

Since the questions asked were necessarily aimed at capturing retrospective events, the period of therapy was limited to the last six months before the interview to avoid falsifications by memory gaps of patient owners [41]. Scientific studies show that the memory on which a retrospective survey relies can be distorted over time; important events and details can be completely forgotten [42, 43]. Figure 2 provides an overview of the data collection process.

### Participants

The survey was completed for 2909 pet bird patients from diverse bird groups, predominantly parrots and parakeets (74.4%) and ornamental fowl (16.1%). We did not impute missing values due to item nonresponses to these pet bird demographics (e.g., some owners did not know the subgroup to which their parrot or parakeet belonged or the sex of the bird).

A total of 2581 bird owners participated in the survey (2017 females, 518 males, 28 did not wish to answer, 18 missing values), with the majority of bird owners between 30 and 44 years (33.8%) and 45–59 years (37.1%) of age (Table 2). Additional descriptive data regarding their sociodemographic characteristics, such as gender, age, domicile, vocational training, employment status, net household income, and history of bird ownership, can be found in Table 2. The number of birds per participant varied: 9.5% of the participants owned only one bird, 17.3% owned two, and 73.2% had three or more pet birds. Please note that, according to animal protection regulations, the birds of most species included in this investigation should not be kept as single birds (with the exception of raptors and owls, who constitute a small group (*N* = 44) in our sample; see Table 3). Note that two groups of bird owners were ineligible for inclusion in the adherence measurement. First, the participants had not visited a veterinarian with their bird in the past six months (*N* = 917). Second, the participants had visited a veterinarian with their bird in the past six months but did not subsequently treat their pet at home (*N* = 219, Fig. 2).

**Table 2:** Bird owner demographics

Total	*N* (relative frequency)
	2581 (100%)
What is your gender?	
Male	518 (20.1%)
Female	2017 (78.1%)
Do not wish to answer	28 (1.1%)
Missing value	18 (0.7%)
Age	
Under 18	30 (1.2%)
18–29	480 (18.6%)
30–44	873 (33.8%)
45–59	958 (37.1%)
60–74	204 (7.9%)
75–89	11 (0.4%)
Over 89	25 (1.0%)
Missing value	0 (0.0%)
Country	
Germany	2383 (92.3%)
Austria	89 (3.4%)
Switzerland	44 (1.7%)
In another country, namely:	35 (1.4%)
Missing value	30 (1.2%)
Domicile	
Large city	412 (16.0%)
Edge or suburb of a large city	402 (15.6%)
Medium-sized or small town	784 (30.4%)
Village in the country	839 (32.5%)
Isolated farmstead or house in the country	104 (4.0%)
Missing value	40 (1.5%)
Vocational training	
No vocational training	278 (10.8%)
Professional qualification	1527 (59.1%)
University degree	562 (21.8%)
Missing value	214 (8.3%)
Employment status	
Working full-time	1129 (43.7%)
Working part-time	407 (15.8%)
Self-employed	245 (9.5%)
Semiretired	17 (0.7%)
Working occasionally	136 (5.3%)
One-euro-job	9 (0.3%)
Working occasionally or on an irregular basis	20 (0.8%)
Doing vocational training/an apprenticeship	39 (1.5%)
Retraining	14 (0.5%)
Voluntary social or ecological year/Federal voluntary service	4 (0.2%)
Maternity leave, educational leave, parental leave, or other leave	58 (2.2%)
Not working	444 (17.2%)
Missing value	59 (2.3%)
Net household income	
Less than 1000 Euro	221 (8.6%)
1000 to under 2000 Euro	547 (21.2%)
2000 to under 3000 Euro	477 (18.5%)
3000 to under 4000 Euro	368 (14.3%)
4000 to under 5000 Euro	209 (8.1%)
5000 Euro and above	181 (7.0%)
No information provided	528 (20.4%)
Missing value	50 (1.9%)
Bird breeding	
I am a commercial breeder	9 (0.4%)
I am a hobby breeder	511 (19.8%)
I do not breed birds	2052 (79.5%)
Missing value	9 (0.3%)
Number of years keeping birds	
0–1	183 (7.1%)
2–5	570 (22.1%)
6–9	307 (11.9%)
10–19	622 (24.1%)
20–29	388 (15.0%)
30–39	286 (11.1%)
40–49	166 (6.4%)
>=50	50 (1.9%)
Missing value	9 (0.4%)
Number of birds currently kept	
I have exactly ONE bird	245 (9.5%)
I have exactly TWO birds	445 (17.2%)
I have THREE OR MORE birds	1887 (73.1%)
Missing value	4 (0.2%)

**Table 3:** Characteristics of the birds receiving treatment

	*N* (relative frequency)			
Bird groups				
Parrots and parakeets (e.g. budgerigar, Fischer’s lovebird, amazon .)	2164 (74.4%)			
Softbills and lories (e.g. mynah, rainbow lorikeet, blackbird .)	21 (0.7%)			
Finches (e.g. domestic canary, zebra finch, Gouldian finch .)	160 (5.5%)			
Raptors and owls	44 (1.5%)			
Pigeons and doves	39 (1.3%)			
Ornamental fowl (e.g. chicken, duck, quail.)	468 (16.1%)			
Ratites (e.g. ostrich .)	1 (0.0%)			
Other	10 (0.3%)			
Do not know	2 (0.1%)			
Total	2909			
Parrot and parakeet subgroups				
Budgerigars	928 (42.9%)			
Grey parrots	378 (17.5%)			
Cockatiels	265 (12.2%)			
Amazons	148 (6.8%)			
Fischer’s lovebird/agapornids	94 (4.3%)			
Cockatoos	48 (2.2%)			
Aras	36 (1.7%)			
Other parrots	123 (5.7%)			
Other parakeets	144 (6.7%)			
Total	2164			
Sex				
Female	1337 (46.0%)			
Male	1420 (48.8%)			
Unknown	152 (5.2%)			
Total	2909			
Age (in years)				
0–1	441 (16.9%)			
2–5	1108 (42.4%)			
6–9	415 (15.9%)			
10–19	390 (14.9%)			
20–29	150 (5.7%)			
30–39	75 (2.9%)			
40–49	28 (1.1%)			
>=50	7 (0.3%)			
Total	2614			
Bird properties as reported by owners*	Mean (SD)	Min	Max	Total
Tameness	3.32 (1.417)	1	5	2854
Sensitivity to stress	2.7 (1.231)	1	5	2787
Nervousness	2.58 (1.217)	1	5	2784
Aggression toward bird keeper	1.36 (0.829)	1	5	2780
Aggression toward other people	1.6 (1.058)	1	5	2789

*measured on a five-point Likert scale: “strongly disagree” (1) to “strongly agree” (5)

The overall veterinary medication adherence index was calculated from 1377 overall therapies for pet bird patients, and the single veterinary medication adherence indices were calculated from a total of 2278 single treatments.

### Data analysis

Descriptive statistics such as the means and standard deviations of the variables of interest were calculated via STATA (STATA Corp LLC, version 18.0; College Station, Texas, USA).

Regarding validation, please note recent advances in distinguishing indices from scales [38] (see Chap. 7). In particular, whereas in a scale, a shared underlying cause for the responses to the items seems plausible, in indices, it seems implausible. Whereas in a scale, the correlation between any pair of items is substantial, in indices, the correlation between some or all item pairs is weak. As a consequence, while scale items can be reduced to a small group of clusters (e.g., by factor analysis) that explain a substantial portion of the total reliability in the item set, index items cannot be reduced in this way, e.g., in our adherence indices, the three items total refusal, change in dosage, and change in the time of treatment share no underlying common cause. Rather, different causes, such as a lack of trust in the diagnosis of the veterinarian, worsening of the bird’s state of illness or employment outside of the home, cause nonadherence to the medication of the bird keeper. The correlations between these pairs of items will be small, and a factor analysis cannot reduce the items to a small group of clusters.

The validity of the measures used in this investigation was confirmed through several tests. Three types of validity were considered [38] (see Chap. 7). Content validity was determined by having the indices reviewed and refined on the basis of recommendations from individuals with expertise in veterinary medicine and instrument development. To test construct validity, this study had to reconsider important aspects related to special situations in veterinary medicine. We were not able to validate our indices against objective measures of adherence, such as electronic monitoring of medication or pharmacy records (which are not available for the medication of birds in Germany) or measures of biomarkers or clinical outcomes (which were not applicable because of the anonymity of the questionnaire). We could not correlate our results with those of other indices because other medication adherence indices were not available for birds. Owing to the lack of objective measures and other indices to be used as the gold standard or for comparison, the sensitivity and specificity of our indices could not be calculated. Finally, construct validity was addressed through the development of items with theoretical and empirical significance for different illnesses and their medication in human medicine. In addition, we used a group comparison approach to address criterion validity. It is well known that birds may fly away and flee from treatment. Catching and fixing a bird for treatment is stressful for the bird, the veterinarian and the bird keeper [44]. However, bird keepers with difficulties in catching a bird should be less adherent to the prescribed therapy if that treatment requires catching and fixing. We created artificially constituted groups defined by whether the bird is caught easily by the bird keeper or not. As expected, bird keepers who agreed with the item “It was easy for me to catch this bird” presented a weakly significant and negative correlation (^#^*p* < 0.10, β = -0.075) in a multivariate linear regression on SVMAI-4 for the oral application type directly into the beak, which means that bird keepers who find it easy to catch their bird are more adherent.

## Results

Almost 80% of the participants in the online survey were female and did not breed birds. Most of the study participants were middle-aged, were living in a medium-sized or small town or village, had professional qualifications, and were working full-time or part-time (Table 2).

Approximately three quarters of the participants answered the questionnaire as owners of a parrot or a parakeet. Budgies, gray parrots and cockatiels represented the largest proportion in descending order. All groups of birds are presented in Table 3.

### Overall treatment characteristics

The following information relates to 1480 overall therapies for pet birds (Table 4). In total, 61.5% of the study participants had completed bird treatment at the time of the survey, whereas 35.4% of the participants had not yet finished bird therapy (implementation phase). At least 3.1% (*n* = 54) stated that they did not know whether the therapy had been completed or not.

**Table 4:** Treatment/therapy characteristics

	*N* (relative frequency)
How often has the bird been to the vets?	
1–2 times	1397 (73.4%)
3–8 times	438 (23.0%)
More than 8 times	61 (3.2%)
Do not know	8 (0.4%)
Total	1904
Primary symptom	
No symptom (health check/preventative examination)	206 (10.9%)
Shortness of breath	204 (10.8%)
Movement disorder/changes to wings and/or legs (lameness, broken bone)	192 (10.1%)
Lethargy, loss of appetite, emaciation	178 (9.4%)
Changes to feathers and skin, itching	137 (7.2%)
Changes to feces/urine	130 (6.9%)
Retching, vomiting	123 (6.5%)
Changes to beak/cere	97 (5.1%)
Changes to eyes/visual disorder	63 (3.3%)
Seizures, trembling, coordination disorder	52 (2.7%)
Swollen abdomen	43 (2.3%)
Behavioral disorder	15 (0.8%)
None of the symptoms listed here, but rather:	446 (23.5%)
I do not remember	8 (0.4%)
Total	1894
Treatment at home	
Yes	1479 (85.3%)
No	255 (14.7%)
Total	1734
Treatment completed	
Yes	1065 (61.5%)
No	612 (35.4%)
Do not know	54 (3.1%)
Total	1731
Number of types of treatment	
1	759 (55.0%)
2	410 (29.7%)
3	160 (11.6%)
4	43 (3.1%)
5	8 (0.6%)
7	1 (0.1%)
Total	1381
Type of treatment	
Giving the bird medication with the drinking water	
Stated	419 (28.3%)
Not stated	1061 (71.7%)
Giving the bird medication with the food	
Stated	269 (18.2%)
Not stated	1211 (81.8%)
Giving the bird medication straight into its beak	
Stated	925 (62.5%)
Not stated	555 (37.5%)
Injecting the bird	
Stated	35 (2.4%)
Not stated	1445 (97.6%)
Treating the bird externally with an ointment or a spray	
Stated	196 (13.2%)
Not stated	1284 (86.8%)
Treating the bird with an inhaler	
Stated	147 (9.9%)
Not stated	1333 (90.1%)
Changing/checking the dressing at certain intervals	
Stated	58 (3.9%)
Not stated	1422 (96.1%)
Giving other food	
Stated	160 (10.8%)
Not stated	1320 (89.2%)
Keeping it in a different way to before	
Stated	96 (6.5%)
Not stated	1384 (93.5%)
In my case, the type of treatment was different, namely:	
Stated	192 (13.0%)
Not stated	1288 (87.0%)
I do not remember	
Stated	5 (0.3%)
Not stated	1475 (99.7%)
Total	1480

Multiple answers were possible when describing the individual therapy of the bird. As shown in Table 4, most cases included oral medication directly into the beak (62.5%), followed by medication by drinking water (28.3%) and medication with food (18.2%). Additional treatment recommendations included the following: an external ointment or a spray 13.2%; a food/diet change of 10.8%; the use of an inhaler of 10.8%; improving the bird’s keeping condition 6.5%; checking the dressing 3.9%; and injecting 2.4%. The types of treatment in the questionnaire were designed to cover as many application forms and treatment methods as possible. However, given the breadth of treatment options, it was also possible to answer the question in an open-text format. 13% of the participants used this opportunity. These treatments could not be included in the therapy adherence index if it was not possible to recode the given answer in the previous list of treatments.

Table 4 also shows how many types of treatment the study participants should carry out on their bird at the same time. Exactly 55% of the bird keepers, the largest group, used only one treatment type. In 29.7% of the cases, two types of treatment were carried out. A total of 11.6% of treatments refer to three distinct types of bird therapy. Furthermore, 3.1% stated four types of treatment, and 0.6% referred to 5 types of treatment at the same time. In one case (0.1%), seven types of bird therapy should be implemented at the same time. In addition, Table 4 shows the distribution of the primary symptoms (as reported by the bird keeper; multiple answers were possible) and how often the bird had been to the vets.

### Characteristics of single types of treatment

With respect to each single type of treatment, we asked questions pertaining to the bird owner’s knowledge about the prescribed therapy for the bird patient (indication, dosage, time point, frequency, duration), one question pertaining to the bird owner’s judgment of the practicability of the prescribed therapy (difficulty), and questions pertaining to the bird owner’s lack of adherence to the therapy (change in the type of treatment, total refusal, early discontinuation, change in dose, change in the time of treatment).

With *n* = 925, oral application directly into the beak was the most often prescribed therapy in our sample. A total of 1.4% of the bird owners using this oral medication did not know, 3.9% only partially knew about the indication of the therapy, 2.8% did not know, 2.8% only partially knew the dosage, 3.7% did not know, and another 2.1% only partially knew the time of treatment. A total of 48.3% of the participants in this group stated that they should give their birds oral medication twice a day. A total of 42.8% of the owners thought that they should give the medications once a day, and 6.9% of the owners thought that the medication should be administered three or more times a day. The duration of oral therapy straight into the beak varied: 44.5% of the bird owners should continue therapy for 6–14 days, 27% for 15 days or longer, 17.5% for 2–5 days and 0.7% for one day. A total of 9.4% of bird owners should provide their bird with oral medicines for a lifetime. A total of 30.8% of the participants who were supposed to give their birds oral medication directly into the beak indicated agreement or complete agreement on the 5-point Likert scale that they had difficulty implementing the therapy. By far, the greatest difficulties were observed with respect to the time of treatment: a total of 15.1% of the bird owners stated that it was not always possible for them to carry out oral therapy at the recommended time. 13% independently decided to continue treating the bird in a different way without discussing this with their veterinarians. In 8.5% of the bird owners in this group of oral medications directly into the beak, poor adherence to the treatment was indicated by failure to adhere to the drug dosage, and 8.4% stopped the therapy of their bird. A total of 4.9% could not implement this oral form of application on their bird at all. In Tables 5 and 6, we present these details on each type of treatment as well as summary data on the types of treatment.

**Table 5:** Attributes of all types of treatment and the four most common types of treatment

	All types of treatment	Oral application straight into the beak	Drinking water application	Feed application	Cutaneous application
Bird owner’s knowledge about prescribed therapy for the bird patient					
	*N* (relative frequency)	*N* (relative frequency)	*N* (relative frequency)	*N* (relative frequency)	*N* (relative frequency)
Indication					
Yes	2175 (94.4%)	872 (94.7%)	374 (88.8%)	256 (94.5%)	192 (98.5%)
No	28 (1.2%)	13 (1.4%)	8 (1.9%)	4 (1.5%)	1 (0.5%)
Partly	101 (4.4%)	36 (3.9%)	39 (9.3%)	11 (4.1%)	2 (1.0%)
Total	2304	921	421	271	195
Dosage					
Yes	2046 (92.7%)	870 (94.4%)	390 (92.4%)	256 (94.5%)	169 (86.7%)
No	76 (3.4%)	26 (2.8%)	19 (4.5%)	4 (1.5%)	12 (6.2%)
Partly	86 (3.9%)	26 (2.8%)	13 (3.1%)	11 (4.1%)	14 (7.2%)
Total	2208	922	422	271	195
Time point					
Yes	1865 (93.8%)	868 (94.2%)	388 (92.2%)	256 (94.8%)	184 (94.4%)
No	85 (4.3%)	34 (3.7%)	27 (6.4%)	10 (3.7%)	4 (2.1%)
Partly	39 (2.0%)	19 (2.1%)	6 (1.4%)	4 (1.5%)	7 (3.6%)
Total	1989	921	421	270	195
Frequency (per day)					
Once	1176(57.2%)	396 (42.8%)	344 (81.3%)	222 (81.9%)	80 (41.0%)
Twice	633 (30.8%)	447 (48.3%)	43 (10.2%)	27 (10.0%)	63 (32.3%)
3 times or more	132 (6.4%)	64 (6.9%)	15 (3.5%)	4 (1.5%)	31 (15.9%)
Different, namely:	105 (5.1%)	15 (1.6%)	21 (5.0%)	17 (6.3%)	20 (10.3%)
I do not remember	9 (0.4%)	3 (0.3%)	0 (0.0%)	1 (0.4%)	1 (0.5%)
Total	2055	925	423	271	195
Duration					
One day	18 (0.8%)	6 (0.7%)	2 (0.5%)	0 (0.0%)	6 (3.1%)
2–5 days	366 (15.9%)	161 (17.5%)	79 (18.9%)	28 (10.3%)	46 (23.7%)
6–14 days	827 (35.9%)	410 (44.5%)	179 (42.7%)	61 (22.5%)	74 (38.1%)
15 days or longer (but not for life)	611 (26.5%)	249 (27.0%)	91 (21.7%)	89 (32.8%)	50 (25.8%)
The therapy will be for life	443 (19.2%)	87 (9.4%)	58 (13.8%)	87 (32.1%)	13 (6.7%)
I do not remember	37 (1.6%)	9 (1.0%)	10 (2.4%)	6 (2.2%)	5 (2.6%)
Total	2302	922	419	271	194
Bird owner’s judgment of the practicability of the prescribed therapy					
I had difficulties					
1. I totally disagree	1288 (55.9%)	334 (36.2%)	340 (81.0%)	225 (83.3%)	96 (49.2%)
2.	301 (13.1%)	150 (16.3%)	28 (6.7%)	14 (5.2%)	32 (16.4%)
3.	279 (12.1%)	155 (16.8%)	21 (5.0%)	18 (6.7%)	26 (13.3%)
4.	215 (9.3%)	131 (14.2%)	15 (3.6%)	9 (3.3%)	22 (11.3%)
5. I completely agree	220 (9.6%)	153 (16.6%)	16 (3.8%)	4 (1.5%)	19 (9.7%)
Total	2303	923	420	270	195
Bird owner’s lack of adherence to therapy					
Change in the type of treatment					
Correct	320 (13.9%)	120 (13.0%)	68 (16.3%)	33 (12.2%)	24 (13.3%)
Incorrect	1976 (86.1%)	801 (87.0%)	350 (83.7%)	238 (87.8%)	171 (87.7%)
Total	2296	921	418	271	195
Total refusal					
Correct	80 (3.5%)	45 (4.9%)	11 (2.6%)	7 (2.6%)	4 (2.1%)
Incorrect	2223 (96.5%)	878 (95.1%)	411 (97.4%)	262 (97.4%)	191 (97.9%)
Total	2303	923	422	269	195
Early discontinuation					
Correct	170 (7.7%)	74 (8.4%)	40 (9.8%)	12 (4.6%)	7 (3.7%)
Incorrect	2045 (92.3%)	804 (91.6%)	368 (90.2%)	249 (95.4%)	184 (96.3%)
Total	2215	878	408	261	191
Change in dose					
Correct	162 (7.8%)	74 (8.5%)	41 (10.0%)	23 (8.8%)	11 (5.8%)
Incorrect	1904 (92.2%)	801 (91.5%)	368 (90.0%)	238 (91.2%)	180 (94.2%)
Total	2066	875	409	261	191
Change in the time of treatment					
Correct	278 (13.1%)	133 (15.1%)	36 (8.8%)	33 (12.7%)	19 (9.9%)
Incorrect	1847 (86.9%)	745 (84.9%)	373 (91.2%)	227 (87.3%)	172 (90.1%)
Total	2125	878	409	260	191

**Table 6:** Attributes of additional types of treatment

	Change of feed	Inhalation	Change of husbandry	Dressing change/check	Injection
Bird owner’s knowledge about prescribed therapy for the bird patient					
	*N* (relative frequency)	*N* (relative frequency)	*N* (relative frequency)	*N* (relative frequency)	*N* (relative frequency)
Indication					
Yes	154 (96.2%)	145 (98.0%)	94 (97.9%)	56 (96.6%)	32 (94.1%)
No	1 (0.6%)	0 (0.0%)	0 (0.0%)	1 (1.7%)	0 (0.0%)
Partly	5 (3.1%)	3 (2.0%)	2 (2.1%)	1 (1.7%)	2 (5.9%)
Total	160	148	96	58	34
Dosage					
Yes	136 (85.5%)	142 (95.9%)		53 (91.4%)	30 (90.9%)
No	8 (5.0%)	4 (2.7%)		2 (3.4%)	1 (3.0%)
Partly	15 (9.4%)	2 (1.4%)		3 (5.2%)	2 (6.1%)
Total	159	148		58	33
Time point					
Yes		137 (92.6%)			32 (94.1%)
No		9 (6.1%)			1 (2.9%)
Partly		2 (1.4%)			1 (2.9%)
Total		148			34
Frequency (per day)					
Once		81 (54.7%)		28 (48.3%)	25 (71.4%)
Twice		43 (29.1%)		5 (8.6%)	5 (14.3%)
3 times or more		12 (8.1%)		5 (8.6%)	1 (2.9%)
Different, namely:		11 (7.4%)		17 (29.3%)	4 (11.4%)
I do not remember		1 (0.7%)		3 (5.2%)	0 (0.0%)
Total		148		58	35
Duration					
One day	1 (0.6%)	0 (0.0%)	0 (0.0%)	0 (0.0%)	3 (8.8%)
2–5 days	7 (4.4%)	11 (7.4%)	6 (6.2%)	14 (24.1%)	14 (41.2%)
6–14 days	15 (9.4%)	33 (22.3%)	23 (24.0%)	21 (36.2%)	11 (32.4%)
15 days or longer (but not for life)	22 (13.8%)	68 (45.9%)	22 (22.9%)	18 (31.0%)	2 (5.9%)
The therapy will be for life	113 (70.6%)	35 (23.6%)	44 (45.8%)	3 (5.2%)	3 (8.8%)
I do not remember	2 (1.2%)	1 (0.7%)	1 (1.0%)	2 (3.4%)	1 (2.9%)
Total	160	148	96	58	34
Location of injection					
Under the skin					19 (57.6%)
Into the muscle					12 (36.4%)
Different, namely:					2 (6.1%)
Total					33
Bird owner’s judgment of the practicability of the prescribed therapy					
I had difficulties					
1. I totally disagree	105 (66.0%)	99 (66.9%)	55 (57.3%)	20 (34.5%)	14 (41.2%)
2.	27 (17.0%)	22 (14.9%)	12 (12.5%)	9 (15.5%)	7 (20.6%)
3.	10 (6.3%)	11 (7.4%)	16 (16.7%)	13 (22.4%)	9 (26.5%)
4.	11 (6.9%)	9 (6.1%)	9 (9.4%)	8 (13.8%)	1 (2.9%)
5. I completely agree	6 (3.8%)	7 (4.7%)	4 (4.2%)	8 (13.8%)	3 (8.8%)
Total	159	148	96	58	34
Bird owner’s lack of adherence to therapy					
Change in the type of treatment					
Correct	29 (18.4%)	20 (13.6%)	16 (16.8%)	5 (8.8%)	5 (14.7%)
Incorrect	129 (81.6%)	127 (86.4%)	79 (83.2%)	52 (91.2%)	29 (85.3%)
Total	158	147	95	57	34
Total refusal					
Correct	4 (2.5%)	4 (2.7%)	1 (1.1%)	2 (3.4%)	2 (5.9%)
Incorrect	155 (97.5%)	144 (97.3%)	94 (98.9%)	56 (96.6%)	32 (94.1%)
Total	159	148	95	58	34
Early discontinuation					
Correct	9 (5.8%)	13 (9.0%)	8 (8.5%)	3 (5.5%)	4 (12.9%)
Incorrect	146 (94.2%)	131 (91.0%)	86 (91.5%)	52 (94.5%)	27 (87.1%)
Total	155	144	94	55	31
Change in dose					
Correct	6 (3.9%)	5 (3.5%)			2 (6.2%)
Incorrect	149 (96.1%)	138 (96.5%)			30 (93.8%)
Total	155	143			32
Change in the time of treatment					
Correct	16 (10.3%)	25 (17.4%)		8 (14.3%)	8 (25.0%)
Incorrect	139 (89.7%)	119 (82.6%)		48 (85.7%)	24 (75.0%)
Total	155	144		56	32

In the following, we highlight some of these findings. Overall, in 19.2% of the cases, the therapy had to be continued indefinitely, most often for changes in feed (70.6%) and husbandry (45.8%). In 13.9% of the cases, the type of treatment was independently changed by the bird keeper. A total of 18.9% agreed or completely agreed that they had difficulties implementing the therapy. A total of 13.1% reported that they changed the time of treatment, and 7.8% reported that they changed the dose. Only 3.5% completely refused to implement the therapy, and 7.7% discontinued treatment at an early point in time.

### Determinants of adherence

The items measuring the determinants of adherence were evaluated on a five-point Likert scale (“strongly disagree” [1] to “strongly agree” [5]). The mean scores and standard deviations for each item are shown in Table 7. As six items had been reverse coded, they had to be recoded before interpretation.

**Table 7:** Determinants of adherence*

	Mean	SD	Min	Max	Total
Patient owner-related factors					
I was scared that the treatment or medication would have bad side effects/effects on this bird.	1.94	1.379	1	5	1442
Unfortunately, I forgot to give this bird its medication on several occasions or to carry out the treatment.	1.13	0.558	1	5	1440
It was difficult to adhere to the prescribed treatment due to the stresses of everyday life.	1.66	1.166	1	5	1436
Quite honestly, I sometimes did not have the motivation to adhere to the treatment proposed by the vet.	1.25	0.776	1	5	1446
I found it easy to understand my vet’s diagnosis.	4.34	1.219	1	5	1416
I sometimes had doubts about this treatment plan.	1.71	1.273	1	5	1433
I decided for myself whether or not the treatment selected by the vet was suitable for the illness in this bird.	1.87	1.402	1	5	1428
I was scared of injuring this bird with this treatment.	1.94	1.416	1	5	1447
I felt that this bird actually did not have anything serious.	1.29	0.819	1	5	1430
I often found it difficult to adhere to the treatment plan for this bird due to my own illness or physical limitations.	1.14	0.582	1	5	1444
Veterinary health care team and system-related factors					
I felt that the vet’s diagnosis was wrong.	1.47	1.045	1	5	1431
My vet explained the diagnosis, causes and treatment methods such that I was able to carry out treatment at home without any problems.	4.35	1.215	1	5	1435
My vet asked me about my personal circumstances, like working hours and income, and adapted the treatment plan accordingly.	1.77	1.356	1	5	1428
I do not think my vet and his/her team were able to spend enough time on me and this bird.	1.46	1.105	1	5	1442
Condition-related factors					
This bird was so ill that I did not want to subject it to the treatment.	1.24	0.730	1	5	1440
Treatment was made more difficult by the fact that this bird was constantly getting worse.	1.68	1.298	1	5	1437
This bird had already previously had other illnesses that made treatment more difficult.	1.45	1.047	1	5	1437
No effective method of treatment was available for this bird’s illness.	1.84	1.371	1	5	1430
Therapy-related factors					
The treatment was too complicated, or extensive, to adhere to it.	1.24	0.731	1	5	1443
The treatment of this bird simply went on for too long.	1.54	1.105	1	5	1423
The treatment of this bird was made more difficult by the fact that it suffered side effects.	1.26	0.821	1	5	1433
I stopped treating this bird (e.g. with medication) after the treatment was constantly being changed.	1.07	0.425	1	5	1438
Patient-related factors					
It was easy for me to catch this bird.	3.50	1.600	1	5	1428
This bird struggled against being treated.	2.86	1.627	1	5	1439
This bird rejected the treatment by, for example, refusing the drinking water containing the medication or pecking at the dressing (or similar factors…).	1.87	1.371	1	5	1438
I trained this bird to get used to the treatment (e.g. with clicker training).	1.67	1.279	1	5	1404
I got the impression that this bird resented me trying to treat it.	2.52	1.624	1	5	1447
Social and economic factors					
My personal circumstances did not suit the prescribed treatment of this bird.	1.25	0.789	1	5	1445
I have somebody who helped me to treat this bird.	2.91	1.846	1	5	1434

From these data, it becomes apparent that patient-related factors were the most important determinants of nonadherence to bird keepers’ behavior: many bird keepers reported that the bird patient struggled against being treated (mean = 2.86, SD = 1.627), resented the keeper trying to treat it (mean = 2.52, SD = 1.624) or rejected the treatment (mean = 1.87, SD = 1.371). Training the bird to get used to the treatment (e.g., with clicker training) was uncommon (mean = 1.67, SD = 1.279). Importantly, these factors are very specific for veterinary medicine, with the exception of children and patients with dementia or psychiatric illnesses in human medicine, where a caregiver takes over responsibilities for medication [28, 34]. Patient owner-related factors such as fear with respect to bad side effects of the treatment (mean = 1.94, SD = 1.379) and injuring the bird with the treatment (mean = 1.94, SD = 1.416) were highly important.

### Index values for overall and individual adherence indices

The mean scores, standard deviations, skewness, and kurtosis coefficients for our final adherence indices are shown in Table 8. Our overall veterinary medication adherence index (OVMAI-5) ranged from min = 0 (full adherence to therapy) to max = 5 points (full nonadherence). The single veterinary medication adherence indices (SVMAI-4) ranged from min = 0 (full adherence to therapy) to max = 4 points (completely lacking therapy compliance or full nonadherence).

**Table 8:** Index values for the overall index and individual indices

	Mean	SD	Skewness	Kurtosis	*N*
Overall index (OVMAI-5)					
Overall adherence index	0.84	1.247	2.117	7.290	1377
Individual indices (SVMAI-4)					
Oral application straight into the beak	0.59	1.023	2.118	7.084	917
Drinking water application	0.52	0.959	2.139	7.251	413
Feed application	0.46	0.881	2.435	9.195	267
Cutaneous application	0.36	0.736	2.885	13.255	195
Change of feed	0.44	0.819	2.476	10.084	157
Inhalation	0.51	0.921	2.295	8.359	145
Change of husbandry	0.53	0.932	1.389	3.695	95
Dressing change/check	0.50	1.066	2.032	5.967	56
Injection	0.73	1.126	1.754	5.354	33
Total number of treatments					2278

The overall index ranged from min = 0 (full adherence to therapy) to max = 5 points (full nonadherence); individual indices ranged from min = 0 (full adherence to therapy) to max = 4 points (full nonadherence). Given is the mean, standard deviation, skewness and kurtosis of the index values, the number of answers and the totals

The mean values for individual indices (SVMAI-4) revealed that adherence to therapy decreased in the following order: (1) cutaneous application (mean = 0.36, SD = 0.736), (2) change in feed, (3) feed application, (4) dressing change, (5) inhalation, (6) drinking water application, (7) change in husbandry, (8) oral application into the beak, and (9) injection (mean = 0.73, SD = 1.126). All distributions were positively and thus right skewed (skewness coefficient > 0) and leptokurtic (kurtosis coefficient > 3) compared with the normal distribution. This means that, according to their self-assessment, the bird owners were more adherent than nonadherent. This is also reflected in the overall veterinary medication adherence index (OVMAI-5), with a mean of 0.84 (SD = 1.247), which was again positive, right skewed, and leptokurtic compared with the normal distribution.

## Discussion

Medication adherence has been identified as an important aspect of human and animal health. In contrast to human medicine, where research on medication adherence is well established, there is still a large gap in knowledge in veterinary medicine. Medication adherence scales and indices developed to measure adherence in humans cannot easily be transferred to veterinary medicine [34] because medication outside animal clinics or veterinary practices is exclusively in the hands of the animal keeper, which takes responsibility for adherence to a prescribed medication.

To address the lack of medication adherence measures for bird patients, we developed two indices to measure the medication adherence behavior of animal keepers, and we additionally included items on intentional and nonintentional barriers and beliefs about necessities and concerns associated with medication. We asked about the application types prescribed by the veterinarians as well as their knowledge of the dose and application intervals, referring to a recall period of six months. The indices were not constructed to be disease-specific and included acute and chronic diseases. The inclusion criterion for measurement was that treatments had to be performed at home no longer than 6 months before. Consequently, the implementation and discontinuation phases of medication were investigated. The medication used in this study was defined broadly, not only as pharmacologically active drugs but also by changing the dressing and keeping conditions, such as the type of feed. The questionnaires were completed via the internet and without the participation of the veterinarian, who had prescribed the treatment. The indices were used for people from German-speaking countries. The questions and items were thus written in German.

### The indices

Two decisions in the index construction must be discussed from a methodological point of view on the basis of the quality criteria of comparability, accuracy, flexibility, transparency, and completeness [45]. Note that there is often a trade-off between these criteria. First, accuracy conflicted with transparency, and we preferred transparency over accuracy when we decided to prioritize total refusal (i.e., the maximum score was assigned if the patient’s owner had not performed any treatment at all). From our point of view, providing transparency with respect to the fact that a major incident of nonadherent behavior of the bird keeper has happened was more important because it makes both indices (SVMAI-4, OVMAI-5) more credible for users (researchers) and key audiences (practitioners) than preserving accuracy. The accuracy criterion would have required that we give all types of treatment equal weights as long as we have only limited knowledge of the prescribed therapy. Second, the same goal conflict occurred for those applications for which a single dimension of nonadherence did not apply (e.g., a change in dosage is irrelevant to “bandage change” therapy). Irrespective of this, we maintain the range of min = 0 and max = 4 for such applications. Again, we argue that providing transparency is more important than providing accuracy. Both decisions have an overall effect that we preferred for transparency: our indices over nonadherence. This tendency should be kept in mind, as we argue in the limitations section that in our sample, bird keepers who reported a higher degree of nonadherent behavior were likely to be underrepresented. The net effect of these opposing tendencies cannot be calculated on the basis of our data.

Regarding decisions on how to weight adherence behavior, we did not prioritize any dimension of non-adherence besides total refusal (mentioned above) because it was not possible to judge the relative importance of each dimension. Similarly, we decided to give all types of treatment equal weighting, as we did not know whether the veterinarian had prioritized any treatments in the prescription. These weighing decisions were thus based on practical veterinary experiences and theoretical, rather than methodological reasons.

The indices and resulting adherence scores would certainly have been different if we had not awarded the maximum score for complete refusal of prescribed medication. However, we decided not to use a different weighting system for sensitivity analyses, since there are reasonable medical grounds to suggest that omitting a medication component could make the treatment ineffective.

### Pet bird treatment and keeper adherence

The descriptive results revealed a challenging variety of treatment types that veterinarians expected to perform with bird keepers as a consequence of a broad range of diseases. Following the general assumption that medication is beneficial, but without knowledge about the significance of cutoff values, we used adherence values between 0 (full adherence) and 4 or 5 (full nonadherence) to rate the adherence of individual owners and to compare different application types.

The survey revealed that total refusal was not the most widely shown nonadherent behavior; rather, changes in the type of treatment and the time of treatment seemed to be common. Overall, 69% of our participants reported that they had no difficulties in applying the prescribed therapy. IIn addition to these general tendencies there were significant differences between the different types of treatment, which is reflected in the different scores at the level of individual indices (0.36 < = Mean _SVMAI−4 x_ < = 0.73). According to the mean values for the individual indices (SVMAI-4), medication adherence was highest for cutaneous application and medication via feed but was relatively low for peroral medication directly into the beak and injections. Median index values close to 1 suggest generally high adherence; however, these values are assumed to be biased because of social desirability effects and positive self-selection of the sample of participants of the online questionnaire, as is known from other investigations [5, 10]. Nevertheless, the results presented here allow a comparison of different medication application types. Notably, the most frequently prescribed application, the peroral type, which stands for the application of pharmacological drugs directly into the beak of the bird, had the second highest nonadherence value, suggesting a substantial risk of treatment failure. Multiple treatments resulted in an increase in nonadherent behavior, as reflected by the overall OVMAI-5 score (mean _OVMAI−5_ = 0.84).

These results indicate a need for action regarding the application types most commonly used in avian medicine. Veterinarians are advised not only to reduce the use of multiple medications but also to prescribe drugs with application types differing from those peroral into the beak or injection as much as possible. At present, however, there is a lack of drugs suitable for feed, water or even percutaneous application in pet birds. From a medical point of view, there is also an urgent need for research in the development of drugs with good or at least neutral tastes for birds, as has been done, for example, for dogs [46], or with a grainy texture that allows easier application via feed with better acceptance by birds. In addition, the results showed that veterinarians might improve adherence by demonstrating and training restraint and application techniques in birds more extensively to reduce stress for birds and keepers and discussing potential problems associated with medication prescriptions with patient keepers to reach the best agreement [21].

The high relevance of animal patient-related factors as barriers to medication adherence was identified, which is especially important for the most frequent application type, the oral medication directly into the beak. Veterinarians must consider that, unlike dogs and cats, pet birds often are not tame (mean 3.32, SD 1.42; see Table 3), and birds may fly away quite easily and flee from medication. From this perspective, full adherence to a prescribed medication is far from self-evident. Oral therapy directly into the beak is known to be very burdensome, particularly for small pet birds and their keepers, including a risk of becoming hurt by bites or scratches [44], similar to the situation in dogs [22] or cats [47].

With respect to the medical relevance of nonadherence, there is a general agreement that not taking a medication as prescribed is likely to mean that the patient is not receiving the full therapeutic benefit of the medication [12]. For human patients, adherence increases the likelihood of a good clinical outcome by approximately 3-fold and reduces the likelihood of dying from individual diseases by approximately 2–3-fold [48, 49]; however, as reflected by the different definitions of nonadherence used in research [10–12] and veterinary medicine [47, 50], it is difficult or even impossible to exactly determine the consequences of nonadherence for therapeutic outcomes. The effect of nonadherence has been shown to vary depending on the disease, and in some cases, when the prescription is suboptimal or inappropriate and not evidence-based, nonadherence might be neutral or protective [12]. With respect to the unclear relevance of medication adherence for the clinical outcome [11], thresholds defining “good” and “bad” adherence, which are widely used, lack evidence to support them. As stated by World Health Organization [1], “good” and “bad” adherence might not exist because the dose–response phenomenon is a continuous function. The indices developed in our investigation combined the possible effects of the number of doses, time points and intervals of medications, resulting in an abstract index value varying from 0 to 4 or 5. Thus, clinically meaningful thresholds for the adherence scores could not be defined in our study; however, the scores allow for a relative evaluation, with lower scores likely indicating better therapeutic outcomes and highlighting problems with the commonly prescribed oral application method directly into the beak. As described above, our study supports the conclusion that searching for alternative application methods could lead to increased adherence. Further investigations using indices in a clinical setting of single well-defined diseases and treatments would be desirable to test the associations of adherence values with clinical outcomes. Such testing was not possible because the study was designed as an anonymous retrospective online survey. One possible study design to determine clinically relevant cutoffs would be a non-anonymous investigation involving a small group of bird owners and their birds. The owners would document their individual adherence behavior, while the veterinarians would record the clinical course of the diseases, which would be treated according to a known protocol.

### Limitations

There are several limitations associated with this study. We focus on the representativeness and generalizability of the results and on the lack of external validation.

First, a convenience sample was used to generate data — a specific type of nonprobability sample that relies on data collection from population members who are conveniently available to participate in a study. Thus, the sources that had to be relied on, such as in social media pools, contained a heterogeneous population (as shown in Table 2) to recognize a wide range of possible owner‒bird relationships. Since data on the pet bird owner population in Germany is not available, and data on bird owners who visit the vet or treat their birds at home is similarly non-existent, the representativeness of the sample of this study cannot be evaluated. Nevertheless, a major German representative study was used for a conservative estimate of the selectivity of our sample (“German General Social Survey (GGSS) - Allgemeine Bevölkerungsumfrage der Sozialwissenschaften (ALLBUS)”) [51]. The comparison was limited by the use of different categories in the reference data. Compared with the German population, our participants were more often female, younger, and single. Males, people over 60 years of age, and households with children were somewhat underrepresented. The low participation rates of older people can be attributed to the use of an online questionnaire. Furthermore, the participants had a higher level of education than did the general population and were more often employed; however, it is quite possible that the bird-owning population, particularly those who have been to the veterinarian with their bird within the last six months and have consulted to treat their bird at home, differs from the general population in Germany. For the purpose of this study, a heterogeneous sample was needed to identify different types of bird species, primary symptoms, and medications. We have endeavored several ways to ensure that our sample is heterogeneous. For example, the participants differed in their sociodemographic characteristics, and they owned bird species belonging to all groups of pet birds (Table 2). It must be expected that there has been at least one systematic, selective effect—positive self-selection into the sample. The participants who did not regard their pet birds as important enough to report about them were probably missed. Moreover, it is likely that the recruiting methods used here were unable to reach owners who are less interested in bird welfare. Whether the number of birds the participants in these investigations influenced the results of this study remains an open question for further research. Therefore, the degree of adherence shown here applies specifically to the sample used in this survey and cannot be generalized to all German bird owners. In particular, we would expect a greater number of owners with a greater degree of nonadherent behavior in the empirical population of bird owners.

A further reason supports this evaluation: we relied on answers from a self-completion questionnaire in which participants will exhibit a tendency toward replying in ways that are meant to be consistent with their perceptions of the desirability of certain kinds of answers, here the desirability of adherence (social desirability bias). Given the impossibility of drawing a representative sample of bird owners and questioning them, it is difficult to ascertain how different the results would be when a representative sample is used. Due to a lack of information on the bird-owning population, or on owners who visit the vet or treat their birds at home, it was not possible to estimate weights and use them for adjusting. Further tests, as mentioned above, could not be used to validate our social science and veterinary index either. Test-retest reliability could not be used to measure consistency and stability over time because the questionnaire was anonymous, and participants could not be identified.

Due to the methodology used, our results are likely to be biased. Understanding the direction of this bias is key to interpreting the results. In our case, it can be concluded that actual medication adherence is probably lower than that reported by our measurement tool, which may have reported the empirical maximum value. The reporting bias might also be reflected by the right-skewed and leptokurtic distribution of the index values. Nevertheless, the ranking of the different forms of therapy is valid due to the heterogeneous sampling, for example, that application via the beak is associated with lower medication adherence than most other application types. An obvious solution to the problems of self-selection, self-reporting and social desirability bias is to observe people’s behavior directly to elicit information on adherence; however, in contrast to humane medicine, where, in some cases, patients in clinics can be observed when taking their medicine, it is practically impossible to observe the treatment of bird patients in the homes of bird keepers.

Second, our sample included several bird species; however, for some of these species, the number of reported birds was rather small. Even if such bird species fall into a bird group category, which we control for, our results hold for the group and not necessarily for each species. Further studies on single bird species are therefore needed.

Third, in 35.4% of our patients, the treatment of the bird patient had not yet been completed; these patients were referred to the implementation phase, whereas the remaining 64.5% of the patients were treated with medication. Therefore, the data referring to each treatment should not be averaged on the case numbers. It is still possible that during the remaining time of treatment, some nonadherent behavior is shown by the bird keeper. Although it would have been possible to remove the not yet completed treatments from the analysis and calculate descriptive and index values only for the completed treatments, we decided to stick to the larger overall sample.

Fourth, some of the answers rely on what the owners inferred about their birds, e.g., answers pertaining to the condition-related factors (e.g., the answer to the item “Treatment was made more difficult by the fact that this bird was constantly worsening”). Basically, such an inference can be right or wrong, which influences whether the prescribed treatment would still be recommended from a veterinarian’s point of view. Without judgment from the veterinarian, however, it is rather difficult to evaluate some behavior of the bird keeper as adherent or nonadherent. Future research could benefit from additionally including the views of others, e.g., veterinarians or behavioral biologists, and could address the stability of the results. Admittedly, this would be a time-consuming and costly process. In particular, the veterinarians or biologists included in the sample would have to be those who treated or observed the behavior of the bird patient.

On the basis of our descriptive results, we cannot yet infer the reasons for the adherent or nonadherent behaviors of bird keepers. Further analyses allow us to examine the relationship between adherence and other concepts either as a control variable or as a variable of interest.

## Conclusion

Medication adherence is an important aspect of any treatment for an illness, whether for human or for animal patients. If the (animal) patient does not receive the prescribed medication, this can contribute to a partial or complete loss of therapeutic success. In veterinary medicine, tools or indices to measure nonadherence are lacking, leading to limited knowledge on the extent of nonadherence and possible causes. The main aim of this study was to develop indices to measure and assess medication adherence for a particular group of animals, companions or pet birds. The newly developed indices were then evaluated in an online survey of pet bird owners. As a first descriptive step, the indices were explained and justified in detail here. Future work will involve using the indices to estimate effect sizes and to perform multivariate analyses of medication adherence and its determinants, in order to identify the causes of non-adherence.

Nonadherence was defined as the extent to which a bird keeper’s behavior—providing medication, following a diet, and/or executing lifestyle changes—does *not* correspond with agreed-upon recommendations from a veterinarian deduced from the adherence definition of the World Health Organization [1] and expressed as values between 0 and 4 or 5, with 0 representing full adherence. Six dimensions of nonadherence were included: change in the type of treatment, total refusal, early discontinuation, change in dosage, change in the time of treatment, and administration of additional nonprescribed medication. Adherence was measured for individual medication forms (drinking water application (medication applied to drinking water), feed application (mixing with the feed), oral application directly into the beak, injection, cutaneous application, inhalation, bandage change and control, change of feed and/or change of husbandry) and as an overall measure if multiple application forms were prescribed. The indices were integrated into an online questionnaire that also included questions on the bird patient, the bird keeper, and the bird-owner relationship.

The use of these indices may help veterinary scientists identify less difficult treatment types and suggest therapies that may lead to increased adherence by bird keepers. An evaluation of the adherence indices revealed that changes in the type of treatment and the time of treatment seemed to be common nonadherence behaviors. Although 69% of our participants reported that they generally had no difficulties in applying the prescribed therapy, there were significant differences between the different types of treatment, which is reflected in the different scores on the level of individual indices (0.36 < = Mean _SVMAI−4 x_ < = 0.73) and peroral application directly into the beak and injection, resulting in the highest nonadherence values. Furthermore, multiple treatments resulted in an increase in nonadherent behavior, as reflected by the overall score (mean _OVMAI−5_ = 0.84).

Some important questions must be addressed in the future. Veterinarians want to understand the causes of nonadherent behavior. Here, our indices will be used as exploratory tools and in multivariate analyses which are recommended to systematically examine the relationship between nonadherence and other concepts, either as variables or variables of interest, as well as qualitative research, e.g., by conducting interviews with bird keepers who have reported that they changed the type of treatment. In the latter case, we would not only like to know the reasons for the change but also the alternative type of treatment that the bird keepers finally applied. The overall aim is to improve success in bird treatment by educating both sides, bird keepers and veterinary staff, and increasing keeper adherence to veterinary recommendations.

## Supplementary Information

Supplementary Material 1.

## Data Availability

The data generated or analyzed during this study are included in this published article and its supplementary information files or are available from the corresponding author upon reasonable request.

## Abbreviations

OVMAI-5: Overall Veterinary Medication Adherence Index
SD: Standard Deviation
SVMAI-4: Single Veterinary Medication Adherence Index
