# Supplementary material for: Medication adherence in pet bird owners: index development and assessment of various application types via an online questionnaire
Source: BMC Vet Res. 2026 Apr 14;22:232. doi: 10.1186/s12917-026-05457-0 (PMC13085360; doi:10.1186/s12917-026-05457-0)
Supplement: Supplementary file 1 — Supplementary Material 1. [file 12917_2026_5457_MOESM1_ESM.pdf]

Questionnaires

Main questionnaire

The unfiltered version of the questionnaire consisted of 190 questions, including 105 filter questions and a total of 745 variables on 69 "online pages."

Online questionnaire

For reasons of space, the list outputs are only printed with a reference to the possible answers.
→ Filter: the question is only displayed to participants to whom this question applies.

1. Welcome/cover letter

THIS IS A SURVEY FOR BIRD OWNERS WHOSE BIRDS NEED TO BE OR HAVE HAD TO BE TREATED FOR AN ILLNESS.

Dear bird owners,

Thank you for taking the time to complete this survey.

As part of a **doctoral thesis** at Ludwig Maximilian University in Munich, we have developed a questionnaire **on veterinary treatment and the challenges associated with adhering to the respective treatment.**

Help us improve the medical care of your bird by sharing your experiences when your bird was ill and filling out this questionnaire!

Participation in the survey is voluntary. In accordance with the Federal Data Protection Act, your information will of course be treated as strictly confidential and will only be stored and processed **anonymously**.

Please note that it is not possible to go back in the questionnaire, so please answer all the questions on a page before clicking "continue."

It takes about 20-30 minutes to complete.

Thank you for your active participation and enjoy filling out the questionnaire!

If you wish, you can take part in a prize draw at the end of the questionnaire. Twenty fantastic bird calendars for 2018 from the publishers KVH, Ackermann, DuMont, and Korsch will be raffled off. We would like to take this opportunity to thank them for their generous support.

Yours sincerely, Prof. Dr. Rüdiger Korbel (Hospital Director) and

Felica Fontaine (doctoral candidate)

If you have any questions or suggestions, please contact Ms. Fontaine personally at felica.fontaine@campus.lmu.de.

The results of this survey will be published on the website of the "Clinic for Birds, Small Mammals, Reptiles, and Ornamental Fish" at Ludwig Maximilian University in Munich after the doctoral thesis has been published.

2. Introductory questions

2.1. Owner biography

Dear bird owners,

Thank you for participating in this study. We would first like to ask you to answer a few questions about your personal history as a bird owner.

1. How many years have you been a bird owner? Please answer in full years and only in whole numbers. If you do not know the exact number of years, please give an approximate estimate.

- ☐ *Free text*

2. Approximately how many birds have you kept during this time? Please enter whole numbers only. If you do not know the exact number, please give an approximate estimate.

- ☐ *Free text*

3. How would you describe your bird keeping?

- ☐ I am a commercial breeder
- ☐ I am a hobby breeder
- ☐ I do not breed birds

2.2. Selection of the bird

Now we would like to ask you about the bird(s) you currently keep or have kept in the last six months.

4. Have you taken your bird or one of your birds to the vet in the last 6 months?

- ☐ Yes → F5
- ☐ No→ *Questionnaire II*

5. *Filter:* ***Selection of bird; Yes, I have been to the vet***

This questionnaire is designed to be answered for only one bird; please read the following options and **select the one that applies to your situation:**

- ☐ If you **only** have **one bird**, please answer the questionnaire for that bird.

- If you have **several birds**, please choose the bird that you **last took to the vet due to illness** and answer all of the following questions in this questionnaire with answers that apply **specifically to this bird**.
- If you have **two birds** that are **ill at the same time**, please answer the questionnaire for the bird with the more severe symptoms.

2.3. Bird species

You have now **selected a bird**; please answer all of the following questions in this questionnaire **specifically for this bird**.

6. To make it easier to answer the questionnaire, we will now list the *most commonly kept* birds first.

What is your bird called?

- budgie
- Cockatiel
- Congo gray parrot
- Timneh grey parrot
- Blue-fronted Amazon
- Catherine parakeet
- Goat parakeet
- Lovebirds
- Canary
- Zebra finch
- Gouldian finch
- Raised duck
- Warty duck
- Araucana
- Marans
- Silkie chicken
- Brahma
- Feather-footed bantam
- Sussex
- Wyandotte
- Orpington
- Pomeranian goose
- Japanese quail
- My bird is not yet listed here. → F7
- I don't know → F27

7. Which group of birds does this bird belong to?

Please select which group this bird belongs to.

- Parrots and parakeets (e.g., budgerigar, lovebird, Amazon parrot, etc.) → F8
- Soft feeders and lorikeets (e.g., beo, rainbow lorikeet, blackbird...) → F16
- Finches (e.g., canary, zebra finch, gouldian finch...) → F17
- Birds of prey and owls → F18
- Pigeons → F19
- Ornamental poultry (e.g., chicken, duck, quail, etc.) → F20
- Raptors (e.g., ostrich...)→ F25
- I don't know → F26
- Other→ F27

8. *Filter:* ***Parrot groups***

Please select the group to which this bird belongs within the "Parrots and Parakeets" group.

- Amazons → F9
- Macaws → F10
- African Grey Parrots → F11
- Cockatoos → F12
- Cockatiels → *F28 (characteristics of the bird)*
- Lovebirds → *F28 (characteristics of the bird)*
- Lovebirds →*F28 (characteristics of the bird)*
- Budgerigars →*F28 (characteristics of the bird)*
- Other parrots → F13
- Other parakeets → F14
- I don't know → F15

9. *Filter:* ***Amazons***

Now please specify the exact name of this bird within the "Amazons" subgroup.

- Blue-fronted Amazon
- Double-yellow-headed Amazon
- Yellow-naped Amazon
- Yellow-crowned Amazon
- Yellow-cheeked Amazon
- Müller's Amazon
- Panama Amazon or Yellow-fronted Amazon
- Venezuela Amazon
- White-fronted Amazon
- Other →*F27*
- I don't know →*F15*

10. *Filter:* ***Macaws***

Now please specify the exact name of this bird within the "Macaws" subgroup.

- Yellow-breasted Macaw or Ararauna
- Green-winged macaw or dark red macaw
- Scarlet macaw or Arakanga
- Hyacinth Macaw
- Red-billed macaw
- Little Macaw
- Other →*F27*
- I don't know →*F15*

11. *Filter:* ***African Grey Parrot***

Now please give the exact name of this bird within the "African grey parrot" subgroup.

- Congo African Grey Parrot
- Timneh African Grey Parrot
- I don't know → *F15*

12. *Filter:* ***Cockatoos***

Now please enter the exact name of this bird within the "Cockatoos" subgroup.

- Yellow-crested cockatoo
- Yellow-cheeked cockatoo
- Goffin's cockatoo
- Inca cockatoo
- Moluccan cockatoo
- Naked-eye cockatoo
- Cockatiel
- Orange-crested cockatoo
- Galah
- White-crested cockatoo
- Other →*F27*
- I don't know →*F15*

13. *Filter:* ***Other parrots***

Now please specify the exact name of this bird within the subgroup "other parrots."

- ☐ Noble parrot
- ☐ Goldbug parrot or Mayer's parrot
- ☐ Green-winged parrot
- ☐ Maximilian parrot
- ☐ Black-headed parrot or Senegal parrot
- ☐ Rust-capped parrot
- ☐ Sparrow parrot
- ☐ Other →*F27*
- ☐ I don't know →*F15*

14. *Filter:* ***Other parakeets***

Now please specify the exact name of this bird within the subgroup "Other parakeets."

- ☐ Barnard's parakeet
- ☐ Mountain parakeet
- ☐ Bourke's parakeet
- ☐ Glitter parakeet
- ☐ Great Alexandrine parakeet
- ☐ Collared parakeet or lesser Alexandrine parakeet
- ☐ Catherine parakeet
- ☐ King parakeet
- ☐ Pennant parakeet
- ☐ Plum-headed parakeet
- ☐ Rosella
- ☐ Jeweled parakeet
- ☐ Beautiful parakeet
- ☐ Singing parakeet
- ☐ Sun parakeet
- ☐ Jumping parakeet
- ☐ Multicolored parakeet
- ☐ Goat parakeet
- ☐ Lemon parakeet
- ☐ Other →*F27*
- ☐ I don't know →*F15*

15. *Filter:* ***Parrots; I don't know***

You selected "I don't know." Can you find this bird in this list? If you cannot find this bird in this list, please check the last answer option, "No, ...".

- ☐ *All possible answers listed under "Parrots and parakeets," excluding "Other" and "I don't know"*
- ☐ No, I cannot find the bird/birds in the list.

16. *Filter:* ***Soft feeders and lorises***

Now please specify the exact name of this bird within the "Soft feeders and lorises" group.

- ☐ Blackbird
- ☐ Bearded reedling
- ☐ Beo
- ☐ Jackdaw
- ☐ Lori
- ☐ Starling
- ☐ Other →*F27*
- ☐ I don't know →*F26*

17. *Filter:* ***Finches***

Now please specify the exact name of this bird within the "Finches" group.

- ☐ Bullfinch
- ☐ Gouldian finch
- ☐ House sparrow
- ☐ Japanese white-eye
- ☐ Canary
- ☐ Polar siskin
- ☐ Rice finch
- ☐ Long-tailed finch
- ☐ Goldfinch
- ☐ Zebra finch
- ☐ Other →*F27*
- ☐ I don't know →*F26*

18. *Filter:* ***Birds of prey and owls***

Now please specify the exact name of this bird within the group "Birds of prey and owls."

- Bearded owl
- Griffon vulture
- Gyrfalcon
- Ger-Lanner falcon
- Goshawk
- Buzzard
- Red-tailed Hawk
- Saker falcon
- Barn owl
- Snowy owl
- Pygmy owl
- Golden eagle
- Steppe eagle
- Kestrel
- Eagle owl
- Tawny owl
- Peregrine falcon
- White-faced Scops Owl
- Desert buzzard or Harris hawk
- Other →*F27*
- I don't know →*F26*

19. *Filter:* ***Pigeons***

Now please specify the exact name of this bird within the "Pigeons" group.

- Diamond Dove
- Carrier pigeon
- Pigeon breed
- City pigeon
- Fruit dove
- Other →*F27*
- I don't know →*F26*

20. *Filter:* ***Ornamental poultry***

Please select the group to which this bird belongs within the "Ornamental poultry" category.

- Ducks →F21
- Pheasants →*F28 (characteristics of the bird)*
- Geese →F23
- Chickens →F22
- Guinea fowl →*F28 (characteristics of the bird)*
- Peacocks →*F28 (characteristics of the bird)*
- Turkeys →*F28 (characteristics of the bird)*

- Quails →F24
- Other →*F27*
- I don't know →*F26*

21. *Filter:* ***Ducks***

Now please specify the exact name of this bird within the "Ducks" subgroup.

- Cayuga duck
- Runner duck
- Mandarin duck
- Peking duck
- Warty duck
- Other →*F27*
- I don't know →*F26*

22. *Filter:* ***Chickens***

Now please specify the exact name of this bird within the subgroup "Chickens."

- Araucana
- Brahma
- Feather-footed bantams
- Marans
- Orpington
- Rhodelander
- Silkie
- Sussex
- Wyandottes
- Bantam Italian
- Other →*F27*
- I don't know →*F26*

23. *Filter:* ***Goose***

Now please specify the exact name of this bird within the "geese" subgroup.

- Celler goose
- Franconian country goose
- Domestic goose
- Humpback goose
- Pomeranian goose
- Other →*F27*
- I don't know →*F26*
-

24. *Filter:* ***Quail***

Now please specify the exact name of this bird within the subgroup "Quails."

- Japanese quail
- California quail
- Mexican tree quail
- Other →*F27*
- I don't know →*F26*

25. *Filter:* ***Ratites***

Now please specify the exact name of this bird within the "Raptors" group.

- Rhea
- Ostrich
- I don't know →*F26*
- Other →*Q27*

26. *Filter:* ***Name, group I don't know***

You selected "I don't know." Can you find this bird in this list? If you cannot find this bird in this list, please check the last answer option, "No, ...".

- *All possible answers listed under all groups, except "Other" and "I don't know"*
- No, I cannot find the bird(s) in the list.

27. *Filter:* ***Name (species) Other***

You have indicated that you own a bird species that is not listed. Please enter this species below. Please enter only one species.

- *Free text*

<u>2.4. Characteristics of the bird</u>

5 items to be measured on a 5-point Likert scale ([1]):

28. Now we would like to learn about the individual characteristics of this bird. To what extent do you agree with the following statements?

- This bird is tame.
- This bird is sensitive to stress.
- This bird is easily frightened.
- This bird is aggressive towards me.
- This bird is aggressive towards other people.

[1] To collect the data, a 5-point Likert scale with response options ranging from "Strongly disagree (1)" to "Strongly agree (5)" was used here and in the following sections.

2.5. Bird data

29. How old is this bird?

Please enter the age of this bird in whole years. Round up months if necessary. Only whole numbers can be entered.

- ☐ The age of this bird in years is: *Free text* _ _
- ☐ Do not know the age of this bird

2.6. Veterinarian

30. Approximately how often have you taken this bird to the veterinarian or had the veterinarian visit you within the last 6 months?

- ☐ 1-2 times
- ☐ 3-8 times
- ☐ More than 8 times
- ☐ I don't know

3. Illness and treatment

3.1. Aspects of illness and treatment

You have selected a bird that you have had to treat for an illness in the last 6 months. Please now recall the symptoms you noticed in this bird.

31. Can you remember the main symptom[2] of this bird when you had to take it to the vet? Please choose the most severe symptom that you noticed first. Only one answer possible.

- ☐ No symptoms (checkup/preventive examination)→ F44
- ☐ Shortness of breath→ F32
- ☐ Choking, vomiting→ F33
- ☐ Changes in feces/urine→ F34
- ☐ Movement disorder/change in wings and/or legs (lameness, broken bone)→ F35
- ☐ Convulsions, tremors, coordination disorder→ F36
- ☐ Apathy, loss of appetite, emaciation→ F37
- ☐ Plumage and skin changes, itching→ F38
- ☐ Beak/wax skin changes→ F39
- ☐ Eye changes/visual impairment→ F40
- ☐ "Swollen belly"→ F41
- ☐ Behavioral disorder→ F42

[2] Here, respondents were asked about the "leading symptom" (the most noticeable symptom) before the initial visit to the veterinarian; for the sake of simplicity and general understanding, the questionnaire refers to a "main symptom."

- ☐ None of the symptoms listed here, but: *Free text* → F43 *(list of all diseases)*
- ☐ I can't remember→ F43 *(list of all diseases)*

32. *Filter:* ***Shortness of breath***

You have checked "shortness of breath." Can you remember the cause of the symptom "shortness of breath"? Multiple answers possible.

- ☐ Mold mycosis ("aspergillosis")
- ☐ Respiratory tract infection of other causes (e.g., pneumonia)
- ☐ Trauma, accident, injury in the respiratory tract area
- ☐ Heart/circulatory problems
- ☐ Space-occupying growth (tumor) in the abdominal cavity
- ☐ Another illness, namely: *Free text*
- ☐ I don't remember→ F43 *(list of all diseases)*
- ☐ No diagnosis was made

33. *Filter:* ***Gagging, vomiting***

You have checked "Gagging, vomiting." Can you remember the cause of the symptom "gagging or vomiting"? Multiple answers possible.

- ☐ Goiter inflammation (trichomonads/yeasts)
- ☐ Incorrect partner feeding/excessive feeding behavior
- ☐ Other crop disease (e.g., injuries, foreign bodies)
- ☐ Foreign body, tumor (not in the crop), causing gagging/vomiting
- ☐ Megabacteria ("going light syndrome")
- ☐ Bornavirus (PDD, neuropathic glandular stomach dilatation)
- ☐ Kidney disease (uremic vomiting)
- ☐ Poisoning (causing gagging/vomiting)
- ☐ Another disease, namely: *Free text*
- ☐ I don't remember→ F43 *(list of all diseases)*
- ☐ No diagnosis was made

34. *Filter:* ***Change in stool/urine***

You have checked "Fecal/urinary changes." Can you remember the cause of the symptom "Fecal/urinary changes"? Multiple answers possible.

- ☐ Proventricular dilatation disease (PDD, Bornavirus)
- ☐ Megabacteria ("going light syndrome")
- ☐ Stomach infection from another cause
- ☐ Kidney tumor
- ☐ Other kidney disease (causing stool/urine changes)

- Another disease, namely: *Free text*
- I don't remember→ F43 *(list of all diseases)*
- No diagnosis was made

35. *Filter:* ***Movement disorder/change in wings and/or legs***

You have checked "Movement disorder/change in wings and/or legs." Can you remember the cause of the "movement disorder/change in wings and/or legs"? Multiple answers possible.

- Broken bone/dislocation
- Injury (e.g., to muscles or ligaments)
- Change/infection of the joints (arthrosis/arthritis)
- Enlargement/mass in the abdomen (constriction of the sciatic nerve)
- Paw pad ulcer ("bumble foot," pododermatitis)
- Another condition, namely: *Free text*
- I don't remember→ F43 *(list of all diseases)*
- No diagnosis was made

36. *Filter:* ***Cramps, tremors, coordination disorder***

You checked "cramps, tremors, coordination problems." Can you remember the cause of the symptoms "cramps, tremors, coordination problems"? Multiple answers possible.

- Vitamin or mineral deficiency **(**causing cramps, tremors, coordination problems)
- Poisoning **(**causing cramps, tremors, coordination problems)
- Trauma (e.g., concussion, spinal injury, nerve damage, etc.)
- Circulatory disorder
- Infection (e.g., bornavirus or salmonellosis)
- Another illness, namely: *Free text*
- I don't remember→ F43 *(list of all diseases)*
- No diagnosis was made

37. *Filter:* ***Apathy, loss of appetite, weight loss***

You have checked "Apathy, loss of appetite, weight loss." Can you remember the cause of the symptoms "Apathy, loss of appetite, weight loss"? Multiple answers possible.

- *List of all diseases (see above)*
- Another illness, namely: *Free text*
- I don't remember
- No diagnosis was made

38. *Filter:* ***Feather and skin changes, itching***

You have checked "Feather and skin changes, itching." Can you remember the cause of the "feather or skin changes, itching"? Multiple answers possible.

- Skin bacteria
- Skin viruses (e.g., circovirus; psittacine beak and feather disease, PBFD)
- Skin parasites
- Malnutrition, e.g., of vitamins/minerals (causing plumage and skin changes)
- Enlargement (skin tumor, uropygial gland ulcer, feather follicle cyst, etc.)
- Hormonal disorders (causing plumage and skin changes)
- Behavioral/postural errors (e.g., feather plucking)
- Skin injuries
- Other internal diseases (e.g., underwing eczema)
- Another disease, namely: *Free text*
- I don't remember→ F43 *(list of all diseases)*
- No diagnosis was made

39. *Filter:* ***Beak/wax skin changes***

You have checked "beak/wax skin changes." Can you remember the cause of the "beak/wax skin changes"? Multiple answers possible.

- Beak fungus
- Beak viruses
- Parasites of the beak (e.g., mange mites)
- Bacteria of the beak
- Hormonal disorder (causing beak/wax skin changes)
- Beak fracture or injury
- Another disease, namely: *Free text*
- I don't remember→ F43 *(list of all diseases)*
- No diagnosis was made

40. *Filter:* ***Eye changes/vision problems***

You have checked "Eye changes/vision problems." Can you remember the cause of the "eye changes/vision problems"? Multiple answers possible.

- Eye bacteria

- Eye parasites
- Eye viruses
- Disease of the outer eye (eyelids, lacrimal gland, etc.)
- Disease of the inner eye (e.g., cataracts, bleeding in the back of the eye)
- Visual impairment
- Diseases transmissible to humans with eye changes/visual impairment (e.g., psittacosis, tuberculosis, salmonellosis, Newcastle disease)
- Another disease, namely: *Free text*
- I don't remember→ F43 *(list of all diseases)*
- No diagnosis was made

41. *Filter:* ***Swollen abdomen***

You have checked "Swollen abdomen." Can you remember the exact cause of the "swollen abdomen"? Multiple answers possible.

- Enlargement (e.g., egg, tumor, organ enlargement)
- Dystocia/fallopian tube disease
- Another illness, namely: *Free text*
- I don't remember→ F43 *(list of all diseases)*
- No diagnosis was made

42. *Filter:* ***Behavioral disorder***

You have checked "Behavioral disorder." Can you remember the exact nature of this bird's behavioral disorder? Multiple answers possible.

- Aggressive behavior toward animals and humans
- Feather plucking
- "Constant egg laying"
- "Constant feeding"
- Movement disorders (behavioral disorder)
- "Constant screaming"
- Another illness, namely: *Free text*
- I don't remember→ F43 *(list of all illnesses)*
- No diagnosis was made

43. *Filter:* ***I don't remember***

You checked "*I can't remember what the main symptom of my bird was.*" Is the disease on this list?

- *List of all diseases (see above)*
- Another disease, namely: *Free text*
- No, I cannot find the disease in the list.

44. *Filter:* ***No symptoms (check)***

You have checked "*No symptom (check)*". Is the disease found in this list?

- *List of all diseases (see above)*
- Another disease, namely: *Free text →45*
- No, I cannot find the disease in the list. *→45*
- This bird did not have any disease→ *Questionnaire II (directly to OBRS)*

45. Do you know whether this bird's disease is transmissible to humans?

Yes, it is transmissible to humans
No, it is not transmissible to humans
I don't know

<u>3.2. Therapy and form of application/type of treatment</u>

In the following, we would like to learn about the treatment of this bird and the **form of treatment**.

46. Has the treatment of this bird now been completed?

Yes
No→ *Filter: "Note: For clarity, the following questions and statements are phrased in the past tense. Please ignore this if this bird is still undergoing treatment and answer the questions anyway. Thank you very much."*

47. Did you give this bird medication *at home* or treat it in any other way (e.g., by changing its diet/care, changing its bandages, or similar)?

Yes→ *F48*
No→ *Go directly to F58 (Questionnaire III)*

48. *Filter:* ***Yes, treated at home***

What type(s) of treatment should you use for this bird? Multiple answers possible.

I should give this bird...

Administer medication via drinking water→ F49
Sprinkle powdered medication over the food→ F50
Administer medication directly into the beak→ F51

Give injections (not in the beak)→ F52

Treat externally with an ointment or spray→ F53

Treat with an inhalation device→ F54

Change/check the bandage at regular intervals →F55

Give different food →F56

Keep differently than before →F57

In my case, the type of treatment was different, namely: *Free text →F62*

I don't remember →*F62*

---

49. You indicated that you should administer **medication** to this bird **via its drinking water**. Please answer the following questions:

If you were instructed to administer **more than one** medication via drinking water, please answer the questions for the medication that caused you the most problems.

a) **How many times a day** should you add the medication to this bird's drinking water?

- ☐ Once
- ☐ 2 times
- ☐ 3 times or more
- ☐ Other, please specify: *Free text*

b) **How many days** should the drinking water treatment be carried out in total?

One day

2-5 days

6-14 days

15 days or longer (but not lifelong)

The therapy should continue for life

I don't remember

c) Do you know the **reason** for administering this medication?

- ☐ Yes
- ☐ No
- ☐ Partially

d) Do you know the **amount** of medication that should be added to the drinking water?

- ☐ Yes
- ☐ No
- ☐ Partially

e) Did you always know **when** to add the medication to the drinking water?
- ☐ Yes
- ☐ No
- ☐ Partially

f) When treating the drinking water, I decided **to continue treating** this bird in **a different way.**

True

Not true

g) **It was difficult for me to administer medication via drinking water.** *Please rate using a 5-point Likert scale ("strongly disagree" to "strongly agree").*

h) Did you have any problems implementing the treatment via the drinking water?

Yes→ Filter i)

No→ *Filter i)*

i) You indicated that you had problems with the treatment via drinking water. Please tick the answer that applies to you:

I was able to implement this form of treatment...

only partially

not at all→ *Continue with question 62 (or additional application form filters)*

implement despite some difficulties

j) I had to **discontinue** the treatment via drinking water for specific reasons.[3]
- ☐ True
- ☐ Not true

k) I was **unable to adhere to** the dosage for this form of treatment**.**
- ☐ True
- ☐ True

l) I was always able to carry out the treatment via drinking water **at the recommended time.**
- ☐ True
- ☐ True

m) The treatment via drinking water took too long, so I **ended** it **prematurely.**

True

True

---

50. You stated that you should **sprinkle powdered medication on** this bird's **food**. Please answer the following questions:

a) How many times a day should you sprinkle the medication over this bird's food?

- ☐ Once
- ☐ 2 times
- ☐ 3 times or more
- ☐ Other, please specify: *Free text*

b) **How many days** should this treatment be carried out in total?

One day

2-5 days

6-14 days

15 days or longer (but not lifelong)

The therapy should continue for life

I don't remember

c) Do you know the **reason** for administering this medication?

- ☐ Yes
- ☐ No
- ☐ Partially

d) Do you know the **amount** of medication that should be sprinkled on the feed?

- ☐ Yes
- ☐ No

[3] Due **to** an inconsistency in the questionnaire, **only six dimensions of the scale are** mentioned, although there were originally seven, as two dimensions were combined in the statistical analyses because they overlapped in content:
Brief description of the problem: Redundancy of the items "discontinuation" and "shortening of treatment duration."
Solution: Both items are evaluated, but a maximum of one point is awarded. More specifically, one point is awarded if the participant answered (a) D2, (b) D5, or (c) D2 and D5 non-adherently.

 Partially

e) Did you always know **when** to sprinkle the medication over the food?

 Yes
 No
 Partially

f) During this treatment, I decided **to continue treating** this bird in **a different way.**

True
Not true

g) **It was difficult for me to administer medication via feed.** *Please rate using a 5-point Likert scale ("strongly disagree" to "strongly agree").*

h) Did you have any problems implementing the treatment via the feed?

Yes→ Filter i)
No→ *Filter j)*

i) You indicated that you had problems with the treatment via feed. Please tick the answer that applies to you:

I was able to implement this form of treatment...

only partially
not at all→ *Continue with question 62 (or additional application form filters)*
implement despite some difficulties

j) I had to **discontinue** the treatment via feed for specific reasons**.**

 True
 Not true

k) I was **unable to adhere to** the dosage for this form of treatment**.**

 True
 True

l) I was not always able to administer the treatment via the feed **at the**

recommended time.

- True
- True

m) The treatment via feed took too long, so I **ended** it **prematurely.**

True
True

51. You stated that you **should administer medication directly into** this bird's **beak.** Please answer the following questions:

a) How many times a day should you administer the medication directly into this bird's beak?

- Once
- 2 times
- 3 times or more
- Other, please specify: *Free text*

b) **How many days** should this treatment be carried out in total?

One day
2-5 days
6-14 days
15 days or longer (but not lifelong)
The therapy should continue for life
I don't remember

c) Do you know the **reason** for administering this medication?

- Yes
- No
- Partially

d) Do you know the **amount** of medication that should be administered directly into the beak?

- Yes
- No

- Partially

e) Did you always know **when** to administer the medication directly into the bird's beak?

- Yes
- No
- Partially

f) I have decided **to continue treating** this bird in **a different way.**

True
Not true

g) **It was difficult for me to administer medication directly into this bird's beak.** *Please rate using a 5-point Likert scale ("strongly disagree" to "strongly agree").*

h) Did you have any problems administering medication directly into this bird's beak?

Yes→ Filter i)
No→ *Filter j)*

i) You indicated that you had problems administering medication directly into this bird's beak.

Please tick the answer that applies to you:

I was able to implement this form of treatment...

only partially
not at all→ *Continue with question 62 (or additional application form filters)*
implement despite some difficulties

j) I had to **discontinue** treatment with medication administered directly into this bird's beak for specific reasons**.**

- True
- Not true

k) I was unable to adhere to the **dosage** for this form of treatment.

- True
- True

l) I was always able to carry out this form of treatment **at the recommended time**.

- True
- True

m) The treatment directly in this bird's beak took too long, so I **ended** it **prematurely.**

True
True

---

52. You stated that you **should give** this bird **injections (not in the beak).** Please answer the following questions:

a) How many times a day should you inject this bird with medication?

- Once
- 2 times
- 3 times or more
- Other, please specify: *Free text*

b) **How many days** should this form of treatment be carried out in total?

One day
2-5 days
6-14 days
15 days or longer (but not lifelong)
The therapy should continue for life
I don't remember

c) Do you know the **reason** for administering this medication?

- Yes
- No
- Partially

d) Do you know the **amount** of medication you should inject into this bird?

- Yes
- No
- Partially

e) Did you always know **when** to inject this bird with the medication?

- Yes
- No
- Partially

f) During this treatment, I decided **to continue treating** this bird in **a different way.**

True
Not true

g) I should take a medication ...

under the skin
into the muscle
Other, namely: *Free text*
...
(multiple answers possible)

h) **It was difficult for me to inject this bird with medication.** *Please rate using a 5-point Likert scale ("strongly disagree" to "strongly agree").*

i) Did you have any problems giving injections to this bird?

Yes→ Filter j)
No→ *F k)*

j) You indicated that you had problems giving this bird injections. Please tick the answer that applies to you:

I was able to administer this form of treatment...

only partially implement
Not implemented at all→ *Continue with question 62 (or additional application form filters)*
Implement despite some difficulties

k) I had to **discontinue** this form of treatment for specific reasons**.**

- True
- Not true

l) I was unable to adhere to the **dosage** for this form of treatment.

- True
- True

m) I was always able to carry out this form of treatment **at the recommended time**.

- True
- True

n) This form of treatment took too long, so I **ended** it **prematurely.**

True

True

---

53. You stated that you should treat this bird **externally with an ointment or spray**. Please answer the following questions:

a) How many times a day should you treat this bird with an ointment/spray?

- Once
- 2 times
- 3 times or more
- Other, please specify: *Free text*

b) **How many days** should this treatment be carried out in total?

One day

2-5 days

6-14 days

15 days or longer (but not lifelong)

The treatment should continue for life

I don't remember

c) Do you know the **reason** for administering this ointment/spray?

- Yes
- No
- Partially

d) Do you know the **amount** of medication you should apply to this bird?

- Yes
- No
- Partially

e) Did you always know **when** to treat this bird?

- Yes
- No
- Partially

f) During this treatment, I decided **to continue treating** this bird in **a different way.**

True
Not true

g) **It was difficult for me to treat this bird externally with an ointment or spray**. *Please rate using a 5-point Likert scale ("strongly disagree" to "strongly agree").*

h) Did you have any problems treating this bird externally with an ointment or spray?

Yes→ Filter i)
No→ *F j)*

i) You indicated that you had problems with this form of treatment. Please tick the answer that applies to you:

I was able to implement this form of treatment...

only partially
not at all→ *Continue with question 62 (or additional application form filters)*
implement despite some difficulties

j) I had to **discontinue** this form of treatment for specific reasons**.**

True
Not true

k) I was unable to adhere to the **dosage** for this form of treatment.

- True
- True

l) I was always able to carry out this form of treatment **at the recommended time**.

- True
- Not true

m) This form of treatment took too long, so I **ended** it **prematurely.**

True
Not true

---

54. You stated that you should treat this bird **with the aid of an inhalation device**. Please answer the following questions:

a) How many times a day should you treat this bird with an inhalation device?

- Once
- 2 times
- 3 times or more
- Other, please specify: *Free text*

b) **How many days** should this form of treatment be carried out in total?

One day
2-5 days
6-14 days
15 days or longer (but not lifelong)
The treatment should continue for life
I don't remember

c) Do you know the **reason** for treatment with the inhaler?

- Yes
- No
- Partially

d) Do you know the **amount** of medication you should put into the inhaler?

- Yes
- No
- Partially

e) Did you always know **when** to treat this bird with an inhaler?

- Yes
- No
- Partially

f) During this treatment, I decided **to continue treating** this bird in **a different way.**

True
Not true

g) **It was difficult for me to treat this bird using an inhalation device.** *Please rate your response using a 5-point Likert scale ("strongly disagree" to "strongly agree").*

h) Did you have any problems treating this bird with an inhalation device?

Yes→ Filter i)
No→ *Filter i)*

i) You indicated that you had problems with this form of treatment. Please tick the answer that applies to you:

I was able to implement this form of treatment...

only partially
not at all→ *Continue with question 62 (or additional application form filters)*
implement despite some difficulties

j) I had to **discontinue** this form of treatment for specific reasons**.**

- ☐ True
- ☐ Not true

k) I was **unable to adhere to** the dosage for this form of treatment**.**

- ☐ True
- ☐ True

l) I was always able to carry out this form of treatment **at the recommended time**.

- ☐ True
- ☐ True

m) This form of treatment took too long, so I **ended** it **prematurely.**

True
True

---

55. You have indicated that you should **change/check** this bird's **bandage** at certain intervals. Please answer the following:

a) How many times a day should you change/check this bird's bandage?

- ☐ Once
- ☐ 2 times
- ☐ 3 times or more
- ☐ Other, namely: *Free text*

b) **How many days** should this bird wear the bandage in total?

One day
2-5 days
6-14 days
15 days or longer (but not for life)
The treatment should last for life
I don't remember

c) Do you know the **reason** for this type of treatment?

 Yes
 No
 Partially

d) Did you always know **when** you should change/check the bandage?

 Yes
 No
 Partially

e) During this treatment, I decided **to continue treating** this bird in **a different way.**

True
Not true

f) **It was difficult for me to change/check the bandage on this bird at regular intervals.** *Please rate using a 5-point Likert scale ("strongly disagree" to "strongly agree").*

g) Did you have any problems changing/checking the bandage on this bird?

Yes→ Filter h)
No→ *Filter h)*

h) You indicated that you had problems changing/checking the bandage on this bird. Please tick the answer that applies to you:

I was able to perform this type of treatment ...

only partially implement
not at all→ *Continue with question 62 (or additional application form filters)*
implement despite some difficulties

i) I had to **discontinue** this form of treatment for specific reasons**.**

True
Not true

j) I was always able to perform this form of treatment **at the recommended time**.

 True

- True

k) This form of treatment took too long, so I **ended** it **prematurely.**

True
True

---

56. You stated that you should give this bird **different food**. Please answer the following questions:

a) **How many days** should this form of treatment be carried out in total?

One day
2-5 days
6-14 days
15 days or longer (but not for life)
The change in diet should be lifelong
I don't remember

b) Do you know the **reason** for the change in diet?

- Yes
- No
- Partially

c) Do you know **how much** food you should feed this bird?

- Yes
- No
- Partially

d) I have decided **to continue treating** this bird in **a different way.**

True
Not true

e) **It was difficult for me to stick to the change in this bird's diet.** *Please rate using a 5-point Likert scale ("strongly disagree" to "strongly agree").*

f) Did you have any problems changing this bird's diet?

Yes→ Filter g)

No→ *Filter g)*

g) You indicated that you had problems changing this bird's diet. Please check the answer that applies to you:

I was able to implement this treatment...

only partially

not at all→ *Continue with question 62 (or additional application form filters)*

implement despite some difficulties

h) I had to **discontinue** this form of treatment for specific reasons**.**

- True
- True

i) I was **unable to adhere to** the dosage when changing the feed**.**

- True
- True

j) I was able to change the feed **at the recommended time**.

- True
- True

k) The feed change took too long, so I **ended** it **prematurely.**

True

True

---

57. You have indicated that you should **keep** this bird **differently than before**. Please answer the following questions:

a) **How many days** should this change in care last in total?

One day

2-5 days

6-14 days

15 days or longer (but not for life)

The change in posture should last for life

I don't remember

b) Do you know the **reason** for the change in attitude?

- Yes
- No
- Partially

c) When changing the husbandry, I decided **to continue treating** this bird in **a different way.**

True

Not true

d) **It was difficult for me to implement the change in care for this bird.** *Please rate using a 5-point Likert scale ("strongly disagree" to "strongly agree").*

e) Did you have any problems changing this bird's care routine?

Yes→ Filter f)

No→ *Filter f)*

f) You indicated that you had problems changing this bird's husbandry. Please tick the answer that applies to you:

I was able to implement this form of treatment...

only partially implement

not at all→ *Continue with question 62 (or additional application form filters)*

implement despite some difficulties

g) I had to **discontinue** the change in husbandry for specific reasons**.**

- True
- Not true

h) Should this bird be kept alone for its recovery? *Multiple answers possible.*

Yes→ *Filter 57.i)*

No

With visual contact to other birds

Filter 57.i) Yes, this bird should be kept alone.

You have indicated that you should keep this bird alone for its recovery.

Was it possible to keep this bird alone?

Yes
No
Partially

58. Filter: **No, no treatment at home**

Was it not necessary to treat this bird at home?

Yes, it would have been necessary, but I was unable to implement the treatment plan at home.→ F40
No, it was not necessary→ Questionnaire III
I don't know→ Questionnaire III

59. Filter: **Yes, it would have been necessary, but...**

You stated that you were unable to implement the treatment plan for this bird at home.

Did you find a solution in consultation with the veterinarian?

Yes, I took this bird in for inpatient treatment
Yes, I visited my veterinarian regularly for treatment of this bird
☐ No, unfortunately we were unable to find a mutual solution.→ F60

60. Filter: No, unfortunately we were unable to find a mutual solution

You have indicated that you did not find a mutual solution with the veterinarian for the treatment of this bird. Which of the following applies in your case? *(Multiple answers possible)*

☐ I asked someone else for advice→ F61
☐ I am a veterinarian and have come up with a solution myself
☐ I am an expert and have come up with a solution myself
☐ I came up with a solution myself
☐ Other, namely: *Free text*

61. Filter: I asked someone else for advice

You stated that you asked someone other than your veterinarian for advice.

Who was this person? *(Multiple answers possible)*

A friend who owns birds
A bird breeder
Another veterinarian
A human doctor
Other, please specify: *Free text*

3.3. Housing at the time of treatment

62. How was this bird kept at the time of treatment?

Parrots, finches, and soft-eaters:

- ☐ Cage in a room
- ☐ Indoor aviary
- ☐ Separate bird room
- ☐ Indoor aviary with adjoining outdoor aviary
- ☐ Bird room with adjoining outdoor aviary
- ☐ Outdoor aviary without shelter
- ☐ Outdoor aviary with shelter

Birds of prey and owls:

- ☐ Display aviary
- ☐ Breeding aviary
- ☐ Moulting house
- ☐ Keeping on high perches (also round perches and arched perches)
- ☐ Keeping on a pendulum perch
- ☐ Keeping on the block
- ☐ Keeping on the sprinkler
- ☐ Flying wire system

Pigeons:

- ☐ Pigeon loft with attached aviary
- ☐ Pigeon loft in a permanent building
- ☐ Outdoor pigeon loft

Ornamental poultry and ratites:

- Coop without outdoor access
- Coop with outdoor access
- Closed outdoor aviary without shelter
- Enclosed outdoor aviary with shelter
- Free in the garden/pasture
- Other, namely: *Free text*

4. Further questions on compliance and adherence

63. Please now think about the treatment of this bird.

Did the veterinarian discuss the treatment plan with you?

No, he drew up the treatment plan on his own
Yes, we drew up a treatment plan together

4.1. Degree of compliance or adherence

Non-prescribed medication (self-medication) (single global question)

64. Did you administer any additional treatments or medications to this bird *in addition* to the treatment plan discussed?

Yes, namely: *Free text*
No

4.2. Determinants of compliance or adherence

29 items to be measured on a 5-point Likert scale ("strongly disagree" to "strongly agree"):

Classification of items into the five dimensions (determinants) of adherence according to the WHO, adapted to veterinary medicine by the expert panel. Additional determinant: PATIENT-RELATED FACTORS.

65. In the following, we ask you to answer a few questions regarding possible sources of problems during the treatment of this bird:

1) I was afraid that the treatment or medication would have adverse side effects/effects on this bird.
2) Unfortunately, I forgot to give this bird its medication or carry out the treatment several times.
3) It was difficult to adhere to the prescribed treatment due to everyday stress.
4) To be honest, I sometimes lacked the motivation to follow the vet's treatment recommendations.

5) I understood my veterinarian's diagnosis well.
6) I sometimes had doubts about this treatment plan.
7) I decided for myself whether the treatment chosen by the veterinarian was appropriate for this bird's illness or not.
8) I was afraid of hurting this bird with the treatment.
9) I had the feeling that this bird was not really seriously ill.
10) Due to my own illness or physical limitations, I often had difficulty adhering to the treatment plan for this bird.
11) I had the feeling that the vet's diagnosis was incorrect.
12) My veterinarian explained the diagnosis, causes, and treatment methods in such a way that I was able to carry out the treatment at home without any problems.
13) My veterinarian asked me about my personal circumstances, such as working hours and income, and adjusted the treatment plan accordingly.
14) I feel that my veterinarian and his team were unable to devote adequate and sufficient time to me and this bird.[4]
15) This bird was so sick that I didn't want to put it through the treatment.
16) The treatment was made even more difficult by the fact that the bird was getting worse and worse.
17) This bird had previously had other illnesses, which made the treatment even more difficult.
18) There was no effective treatment available for this bird's illness.
19) The treatment was too complex or extensive to adhere to.
20) The treatment of this bird simply took too long.
21) The treatment of this bird was complicated by the fact that it suffered from side effects.
22) I stopped treating this bird (e.g., with medication) after the treatment was changed repeatedly.
23) This bird was easy for me to catch.

24) This bird resisted during treatment.

25) This bird refused treatment, for example by refusing to drink its medication or by chewing on its bandage. (or similar ...)

26) I was able to get this bird used to the treatment (e.g., with clicker training).

27) I had the impression that this bird resented me when I tried to treat it.

28) My personal circumstances were not conducive to the prescribed treatment of this bird.

29) I had someone who helped me treat this bird.

66. Were there any other problems or special circumstances during the treatment of this bird?

No, the questionnaire covered everything.
Yes, namely: *Free text*

67. How much has the treatment of this bird cost so far? This includes: costs of treatment including vet visits, medication, new purchases.

<20 euros
21-50
51-100
101-200
201-500
501-1000
Over 1000 euros

<u>5. Owner-bird relationship scale</u>

21 items to be measured on a 5-point Likert scale ("strongly disagree" to "strongly agree")

68. Now we would like to learn about your personal relationship with your bird. To what extent do you agree with the following statements?

1) I enjoy playing with my bird.
2) I believe that my bird understands me.
3) My bird knows when I am feeling down.
4) I consider my bird to be a friend.
5) My bird is an equal member of my family.
6) Sometimes I wonder what my bird is thinking.
7) I can talk to my bird about anything.

8) My bird is like a child to me.
9) My bird gives my life structure.
10) Owning my bird gives me something to take care of.
11) My bird makes me feel needed.
12) Spending time with my bird distracts me from my problems.
13) My bird makes me feel more balanced and content.
14) It is stressful when my bird is sick and I see it suffering.
15) When my bird is sick, it is my duty to take care of it.
16) I pay attention to my bird's body language.
17) My bird has its own unique personality.
18) My bird is a sensitive creature with its own needs.
19) My bird seeks my company of its own accord.
20) My bird always keeps a little distance from me.
21) Actually, my bird ignores me.

6. Big Five Inventory (BFI-10)

10 items to be measured on a 5-point Likert scale ("strongly disagree" to "strongly agree"):

69. Now, in addition to your bird and your individual relationship with this bird, we would also like to get to know you a little better.

To what extent do the following statements apply to you? Answer as spontaneously as possible. There are no right or wrong answers.

1) I am rather reserved and cautious.
2) I easily trust others and believe in the good in people.
3) I am comfortable, prone to laziness.
4) I am relaxed and don't let stress get to me.
5) I have little interest in the arts.
6) I am outgoing and sociable.
7) I tend to criticize others.
8) I complete tasks thoroughly.
9) I easily become nervous and insecure.
10) I have an active imagination and am imaginative.

7. Owner demographics

Almost done! Now we just need some demographic information about you. Please take a moment to fill this out as well. Thank you!

70. What is your gender?

 Male
 Female

71. When were you born? Please enter your year of birth as a four-digit number.

- *Free text* _ _ _

72. Do you have German citizenship?

 Yes
 No, I have the following nationality: *Free text*
 No

73. What is your marital status?

 Married or in a registered civil partnership and living with your spouse
 Married or in a registered civil partnership and living separately
 Widowed
 Divorced
 Single
 No information

74. What is your highest general education qualification?

 Pupil, attending a full-time general education school
 Left school without a secondary school diploma (elementary school diploma)
 Secondary school diploma (elementary school diploma)
 Secondary school diploma (intermediate school leaving certificate)
 Polytechnic secondary school in the GDR with completion of 8th or 9th grade
 Polytechnic secondary school in the GDR with completion of 10th grade
 Technical college entrance qualification, completion of a technical college
 General or subject-specific university entrance qualification/Abitur (Gymnasium or EOS, also EOS with apprenticeship)
 Abitur obtained through adult education
 Another school qualification, namely: *Free text*

75. What vocational training qualifications do you have? Which items on this list apply to you? (Multiple answers possible)

 Still in vocational training (vocational preparation year, apprentice, intern, student)
 Student attending a vocational college, technical college, or similar
 No vocational qualification and not in vocational training
 Completed vocational training (apprenticeship)

- Completed vocational school training (vocational school, commercial school, preparatory service for middle-level civil service)
- Completed training at a technical college in the GDR
- Completed training at a technical college, master craftsman school, technician school, vocational academy, or technical academy
- Bachelor's degree completed at a (technical) university
- Technical college degree (e.g., diploma, master's degree)
- University degree (e.g., diploma, master's degree, state examination, master's degree)
- Doctorate
- Another professional qualification, namely: *Free text*

76. Which employment situation applies to you? Which of the following applies to you? Please note that employment is defined as any paid activity or activity that generates income.

- Full-time employment
- Part-time employment
- Partial retirement (regardless of whether you are currently working or on leave)
- Marginal employment, €450 job, mini-job
- "One-euro job" (while receiving unemployment benefit II)
- Occasional or irregular employment
- In vocational training/apprenticeship
- In retraining
- Voluntary social or ecological year/federal voluntary service
- Maternity leave, parental leave, or other leave of absence (specify partial retirement under C)
- Not gainfully employed (including: school pupils or students who do not work for money, unemployed persons, early retirees, pensioners without additional income)→ F82 *(additional)*

77. How many people live permanently in your household, including yourself? This household includes all persons who live and manage their finances together here. Please also include all children living in the household.

- One person
- Several persons, namely: *Free text*

78. How many people in your household are under 18 years of age? Only whole numbers can be entered.

- The following number of persons under the age of 18 live in my household: *Free text*
- No persons under the age of 18 live in my household.

79. In which country do you live?

- ☐ Germany→ F79 *(additional)*
- ☐ Austria→ *80*
- ☐ Switzerland→ *80*
- ☐ In another country, namely: *Free text→ 80*

80. *Filter:* ***Germany***

Which state do you live in?

I live in:

- ☐ Baden-Württemberg
- ☐ Bavaria
- ☐ Berlin
- ☐ Brandenburg
- ☐ Bremen
- ☐ Hamburg
- ☐ Hesse
- ☐ Mecklenburg-Western Pomerania
- ☐ Lower Saxony
- ☐ North Rhine-Westphalia
- ☐ Rhineland-Palatinate
- ☐ Saar
- ☐ Saxony
- ☐ Saxony-Anhalt
- ☐ Schleswig-Holstein
- ☐ Thuringia

81. Which of the categories on this list best describes where you live?

- ☐ Large city
- ☐ Outskirts or suburb of a large city
- ☐ Medium-sized or small town
- ☐ Rural village
- ☐ Isolated farmstead or detached house in the countryside

82. This question is about evaluating groups in the population with, for example, high, medium, or low incomes. Therefore, we would like to know: What is the average monthly net income of your household as a whole? The average monthly net income of your household is the sum of wages, salaries, income from self-employment, pensions, or retirement benefits. Please also

include income from public subsidies, income from renting and leasing, assets, housing benefits, child benefits, and other income, and then deduct taxes and social security contributions.

- Less than 150 euros
- 150 to 450 euros
- 451 to less than 850 euros
- 851 to under 1,000 euros
- 1,000 to under 1,250 euros
- 1,250 to under 1,500 euros
- 1,500 to under 1,750 euros
- 1,750 to less than 2,000 euros
- 2,000 to less than 2,250 euros
- 2,250 to less than 2,500 euros
- 2,500 to less than 2,750 euros
- 2,750 to less than 3,000 euros
- 3,000 to less than 3,250 euros
- 3,250 to less than 3,500 euros
- 3,500 to less than 3,750 euros
- 3,750 to less than 4,000 euros
- 4,000 to less than 4,500 euros
- 4,500 to less than 5,000 euros
- 5,000 to less than 5,500 euros
- 5,500 to less than 6,000 euros
- 6,000 to less than 7,500 euros
- 7,500 to less than 10,000 euros
- 10,000 to less than 20,000 euros
- 20,000 and above
- No information

83. *Filter:* ***Employment status***

If you are not employed full-time or part-time: Please indicate which group on this list you belong to.

- Student at a general education school
- University student
- Retired, pensioner, early retirement
- Unemployed
- Permanently disabled
- Housewives/househusbands
- Other, namely: *Free text*

8. Attentive

84. How did you find out about this survey? (Multiple answers possible)

- Veterinary practice/clinic
- Friends/acquaintances
- Social networks (e.g., Facebook)
- Through Lilo, the talking parrot
- Internet forum
- Breeding association
- Other, namely: *Free text*

9. Page for comments

If you have any comments, please feel free to note them here: *Free text*

10. Final page

THANK YOU VERY MUCH FOR YOUR PARTICIPATION!

85. If you own several birds, would you be willing to fill out the questionnaire a second time for a second bird that you have also taken to the vet in the last 6 months?

Yes, I would be happy to fill out the questionnaire again right now for a second bird. → F85

Yes, I would be happy to fill out the questionnaire again at a later time for a second bird → F86

No → Q87

86. *Filter;* ***Fill out again***

→ *Link to shortened questionnaire version*

87. *Filter:* ***Another time***

You have indicated that you would like to fill out the questionnaire again at a later date for a second bird.

We would be very grateful if we could send you a reminder by email in a week's time.

Yes, my email address is: *Free text*

No, I will access the link myself a second time.

11. Competition

If you wish, you can enter a competition to win one of 16 fantastic bird calendars for 2018. To do so, we need your contact details separately from this questionnaire.

We guarantee that your contact details will *not* be used for any other purpose.

If you would like to take part in the prize draw, please click here: *Link to the competition*

Shortened questionnaire "Multiple respondents"

At the end of the "main questionnaire," each participant had the option of completing a shortened version of the questionnaire for another sick bird in their flock. The shortened questionnaire, which was answered by bird owners who wanted to participate in the study for another sick bird, contained largely the same items as the "main questionnaire." Only all personal information was omitted: biography, demographics, and the *"Big* Five personality test." All items that could change individually for another bird had to be answered again. Using a 4-digit code invented by the "multiple respondents" themselves, this data from the first questionnaire could be transferred to the inference statistical evaluation of the shortened questionnaire. The shortened version is not shown here, but can be provided on request.
